# PSMD14 drives lung adenocarcinoma progression through HMMR stabilization and dual activation of TGF-β/Smad and PI3K/AKT/mTOR signaling

**DOI:** 10.3389/fimmu.2025.1720799

**Published:** 2025-12-19

**Authors:** Rui Chen, Shijing Wang, Junping Xie

**Affiliations:** 1 Department of Respiratory and Critical Care Medicine, The Second Affiliated Hospital, Jiangxi Medical College, Nanchang University, Nanchang, Jiangxi, China; 2 Department of Respiratory and Critical Care Medicine, The First People’s Hospital of Jiujiang, Jiujiang, Jiangxi, China

**Keywords:** Capzimin, deubiquitination, HMMR, lung adenocarcinoma, PSMD14

## Abstract

**Background:**

Lung adenocarcinoma (LUAD) represents a major subtype of non-small cell lung cancer with limited therapeutic options. While the ubiquitin-proteasome system has been implicated in cancer pathogenesis, the specific role of deubiquitinating enzymes in LUAD remains incompletely understood. This study investigates the clinical significance and molecular mechanisms of PSMD14, a crucial proteasome subunit, in LUAD progression.

**Methods:**

We analyzed PSMD14 expression patterns across multiple databases including TCGA, GEO, and CPTAC. Functional characterization was performed through *in vitro* and *in vivo* experiments including CCK-8, colony formation, Transwell, and xenograft assays. Molecular mechanisms were elucidated using co-immunoprecipitation, ubiquitination assays, and Western blotting. Drug sensitivity was evaluated using CTRP and PRISM databases, and therapeutic potential was validated with the PSMD14 inhibitor Capzimin.

**Results:**

PSMD14 was significantly overexpressed in LUAD tissues at both mRNA and protein levels, demonstrating excellent diagnostic value (AUC = 0.898) and strong prognostic significance for multiple survival endpoints. Mechanistically, PSMD14 directly interacted with HMMR, removing K63-linked ubiquitin chains to enhance its protein stability. The PSMD14-HMMR axis promoted malignant phenotypes, including proliferation, migration, and invasion. Notably, HMMR overexpression rescued these impaired phenotypes induced by PSMD14 deficiency. PSMD14 high expression correlated with immunosuppressive features and coordinated activation of TGF-β/Smad and PI3K/AKT/mTOR signaling pathways. The PSMD14 inhibitor Capzimin exhibited potent anti-tumor effects *in vitro* and *in vivo*, and combination therapy with the TGF-β inhibitor galunisertib demonstrated enhanced efficacy.

**Conclusion:**

Our findings demonstrate that PSMD14 acts as a key driver of LUAD progression by stabilizing HMMR and consequently activates both the TGF-β/Smad and PI3K/AKT/mTOR signaling pathways. Consequently, the PSMD14-HMMR axis emerges as a promising therapeutic target. Inhibition of PSMD14 exhibited significant anti-tumor efficacy, underscoring its potential for clinical translation in LUAD treatment.

## 1 Introduction

Lung adenocarcinoma (LUAD) represents the most prevalent histological subtype of non-small cell lung cancer, accounting for approximately 40% of all cases and posing substantial challenges in clinical management (1, 2). While significant progress has been made in targeted therapies and immunotherapy, the overall survival rate for advanced-stage LUAD patients remains unsatisfactory, highlighting the critical need to identify novel molecular drivers and therapeutic targets (3–5). The ubiquitin-proteasome system (UPS) has emerged as a crucial regulatory mechanism in cancer pathogenesis, with its components representing promising therapeutic targets (5, 6). Ubiquitination, an essential post-translational modification, orchestrates diverse cellular processes such as proliferation, apoptosis, migration, drug resistance, and tumor development (7–9). Consequently, comprehensive investigation of ubiquitination pathways and their key enzymes in LUAD holds significant clinical relevance for developing innovative treatment strategies. However, the specific functions of deubiquitinating enzymes (DUBs), particularly their regulatory mechanisms and therapeutic potential in LUAD progression, remain incompletely characterized and warrant systematic exploration.

PSMD14 (Proteasome 26S Subunit, Non-ATPase 14), also known as POH1 or RPN11, is an essential Zn²^+^-dependent metalloprotease of the JAMM/MPN+ family. As a core component of the 19S proteasome regulatory particle, it possesses the unique ability to cleave ubiquitin chains from proteasome-bound substrates, playing crucial roles in protein degradation and cellular homeostasis (10–12). Distinct from other deubiquitinating enzymes (DUBs), PSMD14 specializes in cleaving ubiquitin chains from proteasome-bound substrates, a function critical for proteasomal activity. Accumulating evidence has linked PSMD14 to the pathogenesis of multiple human cancers—such as breast cancer, hepatocellular carcinoma, bladder cancer, glioblastoma, and osteosarcoma (13–19) —where it facilitates tumor progression primarily by stabilizing key oncogenic substrates through deubiquitination. Moreover, PSMD14 inhibitors have been reported to suppress tumor metastasis and enhance chemotherapy efficacy (20). Recent studies have revealed that PSMD14 activates specific oncogenic pathways via K63-linked deubiquitination in a context-dependent manner. For instance, Liu et al. reported that in breast cancer, PSMD14 stabilizes FOXM1 through K63-deubiquitination, thereby activating the PI3K/AKT/mTOR pathway (13). Similarly, Yuan et al. demonstrated that in lung cancer, PSMD14 promotes TGF-β1/Smad3 signaling via deubiquitination of Smad3 (21). Although these studies underscore a similar molecular mechanism—whereby PSMD14 stabilizes substrates via K63-deubiquitination to activate specific signaling cascades—the full spectrum of its substrates, pathway interconnectivity, therapeutic targeting potential, and immunomodulatory roles in LUAD remains incompletely understood. This knowledge gap hinders a comprehensive understanding of its oncogenic roles.

Hyaluronan-mediated motility receptor (HMMR), originally identified as a hyaluronan-binding protein, has gained increasing recognition as a multifunctional oncoprotein involved in cell cycle regulation, mitotic spindle formation, and cancer progression (22–24). Beyond its fundamental role in ensuring proper chromosome segregation during mitosis, HMMR participates in various signaling networks including RAS-MAPK pathway and regulates cellular processes such as motility, adhesion, and proliferation. Accumulating clinical evidence demonstrates that HMMR overexpression correlates strongly with poor prognosis across multiple cancer types (25–27). Mechanistically, HMMR promotes malignant phenotypes through diverse mechanisms, including enhancement of cell cycle progression, facilitation of epithelial-mesenchymal transition, and interaction with key oncogenic signaling molecules. Interestingly, both PSMD14 and HMMR have been independently associated with genomic instability and chromosomal aberrations (28, 29), suggesting their potential involvement in common pathogenic processes. However, despite these parallel associations with cancer hallmarks, potential functional interactions between these two molecules in cancer biology remain completely unexplored, presenting a significant knowledge gap in understanding their coordinated roles in tumorigenesis.

In this study, we systematically investigated the clinical significance and molecular mechanisms of PSMD14 in LUAD progression. Our findings demonstrate that PSMD14 is significantly overexpressed in LUAD tissues and correlates with poor patient survival. Mechanistically, we identified HMMR as a novel substrate of PSMD14 and elucidated the molecular basis of their interaction. PSMD14 coordinately activates both the TGF-β/Smad and PI3K/AKT/mTOR pathways, thereby synergistically driving malignant progression in LUAD. Moreover, our work reveals a pivotal role of PSMD14 in the formation of the immunosuppressive tumor microenvironment. Furthermore, we investigated the therapeutic potential of targeting PSMD14 using the specific inhibitor Capzimin, both as monotherapy and in combination with TGF-β pathway inhibition. Our work establishes the PSMD14-HMMR axis as a key driver of LUAD progression and provides compelling evidence for targeting this pathway as a novel therapeutic strategy.

## 2 Materials and methods

### 2.1 Cell lines and human tissues

A panel of human non-small cell lung cancer (NSCLC) cell lines, including H1299, HCC827, H1650, and A549, was obtained from the Cell Bank of the Chinese Academy of Sciences (Shanghai, China). The PC9 and H1975 cell lines were purchased from ServiceBio Co., Ltd. (Hubei, China), while the human normal bronchial epithelial cell line HBE135-E6E7 was acquired from Senbeijia Biotechnology Co., Ltd. (Nanjing, China). A549 and H1299 cells were maintained in DMEM medium (Solarbio, China) supplemented with 10% fetal bovine serum (FBS; ExCell Bio, China) and 1% penicillin/streptomycin (P/S). The remaining NSCLC cell lines (HCC827, H1650, PC9, and H1975) were cultured in RPMI 1640 medium (Solarbio, China) containing 10% FBS and 1% P/S. HBE135-E6E7 cells were grown in Keratinocyte Serum-Free Medium (Zhong Qiao Xin Zhou Biotechnology, Shanghai, China) supplemented with 0.005 mg/mL insulin, 500 ng/mL hydrocortisone, and 1% P/S. All cells were incubated at 37°C in a humidified atmosphere with 5% CO_2_. Each cell line was authenticated by short tandem repeat (STR) profiling.

In addition, twelve pairs of fresh-frozen LUAD tissues and matched adjacent non-cancerous specimens were collected for Western blot analysis. Written informed consent was obtained from all participants before sample and clinical data collection. The study protocol was approved by the Medical Research Ethics Committee of The Second Affiliated Hospital of Nanchang University.

### 2.2 Immunohistochemical staining

Tissue specimens were fixed in 4% paraformaldehyde, embedded in paraffin, and sectioned. Following deparaffinization at 70°C, the sections were cleared in xylene and rehydrated through a graded ethanol series. Antigen retrieval was carried out using EDTA buffer, and endogenous peroxidase activity was quenched by treatment with 3% hydrogen peroxide. The sections were then blocked with 5% goat serum and incubated overnight at 4°C with primary antibodies targeting PSMD14 (1:100; Proteintech, China) and HMMR (1:100; Abways, China). Subsequently, biotin-conjugated secondary antibodies were applied, and signal detection was performed using 3,3′-diaminobenzidine (DAB) as the chromogenic substrate. Finally, the sections were counterstained with hematoxylin, dehydrated through xylene, and examined under a light microscope.

### 2.3 Lentiviral transfection

To generate isogenic models for functional studies, lentiviral particles encoding shRNAs or overexpression constructs for PSMD14 and HMMR were obtained from General Biol Co., Ltd. (Anhui, China). Stable LUAD cell lines with knocked-down or overexpressed PSMD14/HMMR, along with matched controls, were established through lentiviral transduction and subsequent puromycin selection. The specific shRNA sequences used are provided in **Supplementary Table 1**.

### 2.4 Quantitative real-time PCR

Upon reaching 70–90% confluency, LUAD cells were harvested, and total RNA was purified using the RNA-Quick Purification Kit (Esun Bio, China). cDNA was synthesized from the RNA samples with the PrimeScript™ RT Reagent Kit (TaKaRa Bio, Japan). qRT-PCR assays were conducted with TB Green^®^ Premix Ex Taq™ II on a real-time PCR system, and gene expression was quantified by the 2^(−ΔΔCT) method using GAPDH as the endogenous control. Corresponding primer sequences can be found in **Supplementary Table 2**.

### 2.5 Western blotting

Total protein was extracted from both LUAD tissues and cultured cells using lysis buffer. Following quantification with a bicinchoninic acid (BCA) assay kit (Beyotime Biotechnology, China), equal amounts of protein were separated by 10% SDS-PAGE and transferred onto PVDF membranes (Millipore, USA). The membranes were then blocked for 1 hour at room temperature with 5% BSA in TBST. Subsequently, they were incubated overnight at 4°C with specific primary antibodies, followed by a 1-hour incubation with appropriate HRP-conjugated secondary antibodies at room temperature. Protein bands were visualized using an ultrasensitive chemiluminescent substrate (UElandy, China) and captured with a digital imaging system. Relative protein expression levels were determined by densitometric analysis of Western blot bands using ImageJ software, with normalization to the corresponding GAPDH levels. And the original, uncropped blots are provided in the **Supplementary Material**.

### 2.6 Colony formation assay, cell counting kit-8 assay, and EdU incorporation assay

For the CCK-8 assay, LUAD cells were seeded in 96-well plates at a density of 1 × 10³ cells per well in 100 µL of complete medium and allowed to adhere under standard culture conditions. At the indicated time points (0, 24, 48, 72, and 96 hours), 10 µL of CCK-8 solution (GLPBIO, USA) was added to each well. The plates were then incubated at 37°C for 2 hours in the dark. The optical density at 450 nm was subsequently measured using a microplate reader.

For the colony formation assay, LUAD cells were plated in 6-well plates at a low density of 2,000 cells per well and cultured for 10–14 days. The medium was refreshed every 3–4 days. Once visible colonies had formed, with each typically consisting of >50 cells, the cultures were terminated. The colonies were fixed with 4% paraformaldehyde for 30 minutes, gently washed with PBS, and stained with 1% crystal violet for 30 minutes. After thorough rinsing with distilled water and air-drying, the colonies were imaged and manually counted.

For the EdU assay, cell proliferation was assessed using the YF^®^ 594 Click-iT™ EdU Kit (UElandy, China) following the manufacturer’s protocol. In brief, cells were incubated with 10 µM EdU for 2 hours under standard conditions to label replicating DNA. Thereafter, cells were fixed with 4% paraformaldehyde for 15 minutes and permeabilized with 0.5% Triton X-100 in PBS for 20 minutes at room temperature. After washing with PBS, the Click-iT reaction cocktail was applied for 30 minutes in the dark to covalently conjugate the YF^®^ 594 azide to the EdU moiety. Finally, cell nuclei were counterstained with Hoechst 33342 for 10 minutes. Fluorescent images were captured using a fluorescence microscope, and the ratio of EdU-positive (red) cells to total Hoechst-positive (blue) cells was calculated to determine the proliferation rate.

### 2.7 Cytotoxicity assay

The cytotoxicity of Capzimin against LUAD cells was assessed using the CytoTox 96^®^ Non-Radioactive Cytotoxicity Assay (Promega, G1780). After 48 hours of drug treatment, the cell culture supernatants were collected and transferred to a new 96-well plate. Subsequently, 50 μL of the freshly prepared CytoTox 96^®^ Reagent was added to each well, and the plate was incubated in the dark at room temperature for 30 minutes. The reaction was terminated by adding 50 μL of Stop Solution, and the absorbance was measured at 490 nm. The maximum lactate dehydrogenase (LDH) release control was obtained by lysing vehicle-treated cells with the provided 10X Lysis Solution 45 minutes prior to supernatant collection. The percentage of cytotoxicity was calculated as follows: (Experimental LDH Release/Maximum LDH Release) × 100%.

### 2.8 Wound healing assay and transwell assay

Cell migration and invasion were assessed using wound healing and Transwell assays, respectively. For the wound healing assay, LUAD cells were seeded in 6-well plates. Upon reaching 80% confluency, a uniform scratch wound was created in each monolayer using a sterile 200-µL pipette tip. After washing with PBS to remove debris, the cells were cultured in medium containing 3% FBS. Wound areas were photographed at 0, 24, and 48 h under a phase-contrast microscope. The cell migration rate was calculated using the following formula: [1 − (current wound area/initial wound area)] × 100%.

For the Transwell assays, cell invasion was evaluated using Matrigel^®^-coated inserts (BD Biosciences). Briefly, Matrigel^®^ was diluted 1:8 in serum-free medium, and 40 µL was applied to the upper chamber of 8-µm pore inserts. Then, 2×10^4^ cells in 200 µL serum-free medium were added to the upper chamber, while the lower chamber was filled with 700 µL medium containing 20% FBS as a chemoattractant. After 36 h of incubation, non-invading cells were removed, and cells on the lower membrane surface were fixed with 4% PFA, stained with 1% crystal violet, and quantified. The migration assay was performed identically but using uncoated inserts.

### 2.9 Protein stability analysis

Protein stability of HMMR following PSMD14 modulation was evaluated using cycloheximide (CHX; HY-12320, MedChemExpress) chase assays. LUAD cells at 80% confluency were treated with 100ug/mL CHX. Total protein was harvested at 0, 3, 6, 9, and 12-hour post-treatment, and the levels of PSMD14 and HMMR were determined by Western blotting.

### 2.10 Co-immunoprecipitation, ubiquitination assay, and mass spectrometry

For all Co-IP-based experiments, cells were lysed in NP-40 buffer containing protease inhibitors and 20uM MG-132. Cleared lysates were prepared by centrifugation.

Mass spectrometry (MS) for Interactome Identification: Cleared lysates from H1299 cells were immunoprecipitated using an anti-PSMD14 antibody (ab109130, Abcam) conjugated to Protein A/G Magnetic Beads (HY-K0202, MedChemExpress). The bound proteins were separated by SDS-PAGE and stained with Coomassie Blue. Entire lanes were excised, subjected to in-gel tryptic digestion, and the resulting peptides were analyzed by LC-MS/MS (Genechem, Shanghai, China; **Supplementary Table 3**).

Ubiquitination Assay: To assess specific ubiquitination linkages, cells were co-transfected with plasmids encoding HA-tagged wild-type ubiquitin (HA-Ub-WT), lysine 48-only mutant (HA-Ub-K48), or lysine 63-only mutant (HA-Ub-K63). Forty-eight hours post-transfection, cells were treated with 20uM MG-132 for 8 hours to inhibit proteasomal degradation and stabilize ubiquitinated proteins. Cells were then lysed, and immunoprecipitation was performed as described above using the relevant antibodies. The ubiquitination status of HMMR was subsequently assessed by Western blotting using an anti-HA antibody.

### 2.11 Immunofluorescence

Cells grown on glass coverslips were fixed with 4% paraformaldehyde for 30 minutes at room temperature. After three washes with PBS, the cells were permeabilized with 0.2% Triton X-100 in PBS for 10 minutes and blocked with 5% BSA for 1 hour. Subsequently, the cells were incubated overnight at 4°C with primary antibodies diluted in 5% BSA. Following five 5-minute washes with PBS, fluorophore-conjugated secondary antibodies (in 5% BSA) were applied for 2 hours at room temperature in the dark. Cell nuclei were counterstained with DAPI (1 μg/mL) for 5 minutes. Fluorescent images were captured using a suitable microscope.

### 2.12 Flow cytometry

Cell cycle distribution and apoptosis were analyzed by flow cytometry. For cell cycle analysis, cells were harvested 48 hours post-transfection, fixed in 75% ethanol at -20°C overnight, and stained using the Cell Cycle and Apoptosis Kit (UElandy, China) according to the manufacturer’s instructions prior to analysis. For apoptosis assessment, drug-treated cells were stained with the FITC-Annexin V/PI Kit (UElandy, China) for 15 minutes at room temperature in the dark. Samples were analyzed within 1 hour. All flow cytometric data were acquired on a Beckman Coulter CytoFLEX S instrument and processed with CytExpert 2.4 software.

### 2.13 Detection of mitochondrial membrane potential

Changes in mitochondrial membrane potential (ΔΨm) following PSMD14 inhibition were assessed by flow cytometry using the JC-1 fluorescent probe. H1299 and PC9 cells were treated with the PSMD14 inhibitor Capzimin or a vehicle control (0.1% DMSO) for 24 hours. Subsequently, the JC-1 fluorescent probe (Beyotime, Shanghai, China) was employed to detect changes in ΔΨm, and fluorescence was quantified following the supplier’s protocol.

### 2.14 *In vivo* tumor growth

All animal experiments were approved by the Institutional Animal Care and Use Committee of Nanchang Royo Biotech Co., Ltd. (Approval No. RYE2024102802) and conducted in accordance with the AVMA Guidelines for the Euthanasia of Animals (2020). Male BALB/c nude mice (4–6 weeks old) were obtained from Sibeifu Biotechnology Co., Ltd. (Beijing, China). To establish xenograft models, exponentially growing LUAD cells were subcutaneously injected into the right flank of each mouse at a density of 5 × 10^6^ cells (n = 5 per group). To evaluate the antitumor efficacy of Capzimin (AmBeed, USA) and Galunisertib (AmBeed, USA) *in vivo*, tumor-bearing mice were randomly assigned to four treatment groups: vehicle control, Capzimin monotherapy (2.5 mg/kg, i.p.), Galunisertib monotherapy (75 mg/kg, p.o.), and their combination. All treatments were administered once daily for five consecutive days per week over a total of three weeks. Mouse body weight and tumor volume were measured every 5 days following the initiation of treatment. Tumor volume was calculated as (length × width²)/2. At approximately 4 weeks post-inoculation, all mice were humanely euthanized by controlled CO_2_ asphyxiation.

### 2.15 Bioinformatics analysis

All bioinformatics analyses were performed using R (v4.3.3) and public databases. Comprehensive bioinformatic analyses were conducted to elucidate the expression pattern, clinical relevance, and functional implications of PSMD14 in LUAD. These analyses integrated multi-omics data from public repositories, including TCGA, GTEx, and CPTAC, and encompassed assessments of diagnostic performance, survival association, pathway activity, tumor immune microenvironment, and drug sensitivity. Detailed descriptions of the datasets, computational methods, and statistical procedures are provided in the **Supplementary Methods**.

### 2.16 Statistical analysis

A comprehensive statistical approach was employed throughout this study, utilizing both R software (version 4.3.3) for bioinformatic analyses and GraphPad Prism (version 10.1.2) for experimental data analysis. For comparisons between groups, continuous variables were analyzed using Student’s t-test (for parametric data) or the Wilcoxon rank-sum test (for non-parametric data), while one-way ANOVA or Kruskal-Wallis test was applied for multiple group comparisons. Categorical variables were assessed using the Chi-square test or Fisher’s exact test. Correlation analyses were performed using Pearson’s method for normally distributed data and Spearman’s rank correlation for non-normal distributions. Survival outcomes were evaluated through Kaplan-Meier curves with log-rank testing, supplemented by univariate Cox proportional hazards regression where appropriate. Diagnostic performance was quantified by receiver operating characteristic (ROC) analysis, reporting the area under the curve (AUC) with 95% confidence intervals. Continuous data are presented as mean ± standard deviation (SD) unless otherwise specified. Statistical significance was defined as a two-tailed p-value < 0.05, with asterisks denoting hierarchical thresholds: *p < 0.05, **p < 0.01, ***p < 0.001, and ****p < 0.0001; ‘ns’ indicates non-significance.

## 3 Results

### 3.1 PSMD14 is highly expressed and correlates with poor prognosis in LUAD

**Figure 1:**
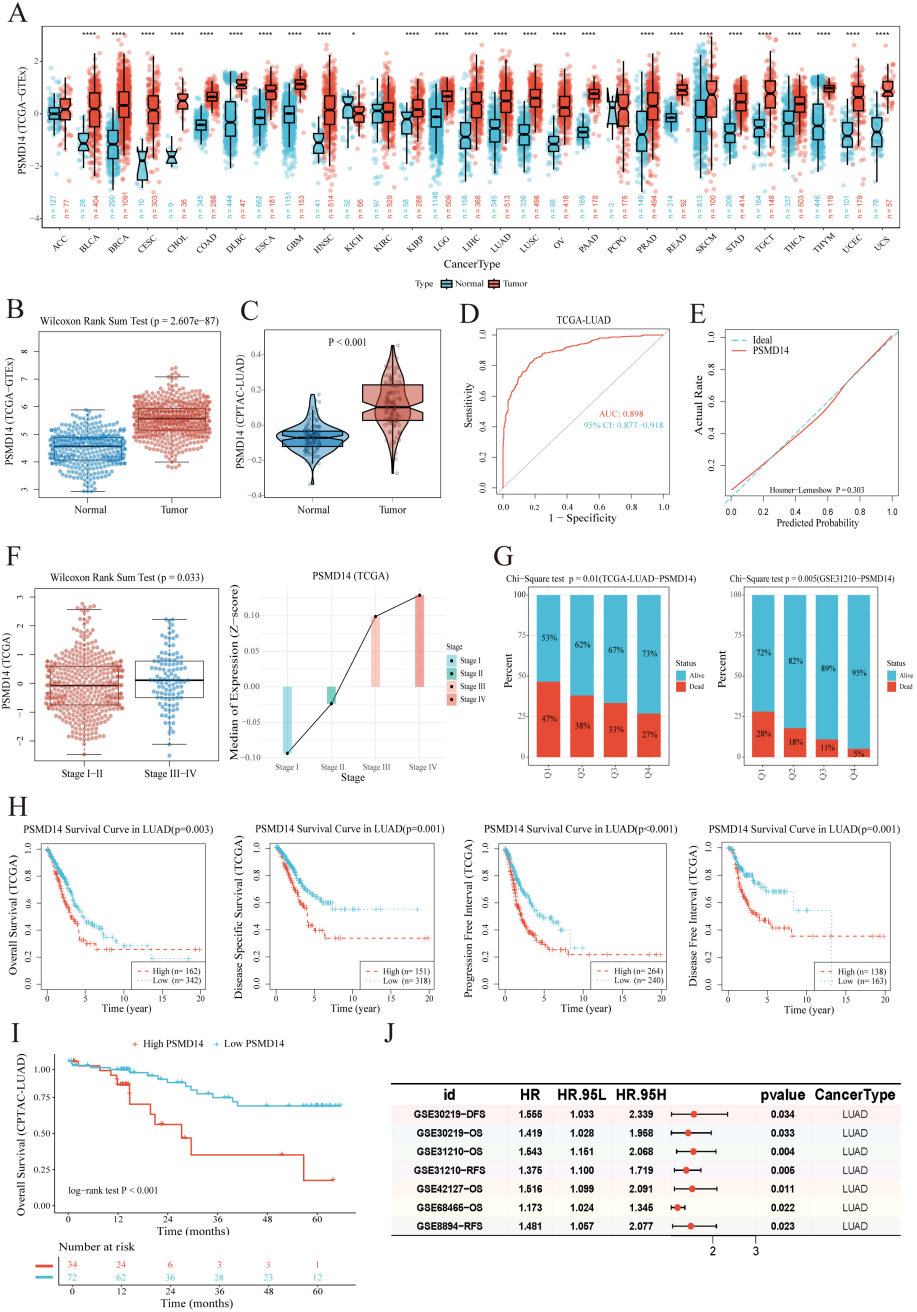
High expression of PSMD14 is associated with poor prognosis in LUAD. **(A)** Comparison of PSMD14 expression between tumor and normal tissues across multiple cancer types (pan-cancer analysis). **(B)** mRNA expression levels of PSMD14 in LUAD and normal tissues from the TCGA and GTEx databases. **(C)** Protein expression levels of PSMD14 in LUAD and normal tissues based on the CPTAC-LUAD dataset. **(D)** Receiver operating characteristic (ROC) curve evaluating the diagnostic value of PSMD14 in LUAD. **(E)** Calibration curve for the PSMD14-based diagnostic model. **(F)** Positive correlation between PSMD14 expression and advanced tumor stage in LUAD patients. **(G)** Distribution of patient survival status across PSMD14 expression quartiles: Q1 (top 25%) to Q4 (bottom 25%). The Q1 group showed a significantly higher number of deceased patients. **(H)** Kaplan–Meier curves comparing overall survival (OS), disease-specific survival (DSS), progression-free interval (PFI), and disease-free interval (DFI) between PSMD14 high- and low-expression groups. **(I)** Kaplan–Meier analysis of OS based on PSMD14 protein expression in the CPTAC-LUAD cohort. **(J)** Forest plot summarizing the association between PSMD14 expression and patient survival across multiple GEO datasets. *P < 0.05, ****P < 0.0001.

PSMD14 demonstrates elevated mRNA expression across multiple cancer types compared to normal tissues (**Figure 1A**). In lung adenocarcinoma (LUAD), PSMD14 is upregulated not only at the transcriptional level (**Figure 1B**) but also at the protein level (**Figure 1C**). The Wilcoxon rank-sum test revealed a significant difference in PSMD14 expression between normal and tumor tissues (P < 0.001), indicating a marked expression disparity between the two groups. More importantly, PSMD14 exhibits significant diagnostic value in LUAD. Receiver operating characteristic (ROC) curve analysis yielded an area under the curve (AUC) of 0.898 (95% CI: 0.877–0.918) (**Figure 1D**), suggesting excellent diagnostic performance. Calibration curve analysis showed a Hosmer–Lemeshow P-value of 0.303 (**Figure 1E**), indicating high consistency between model predictions and observed outcomes. These findings suggest that PSMD14 may serve as a potential biomarker for LUAD with substantial clinical utility.

Furthermore, in LUAD patients, PSMD14 expression was positively correlated with tumor stage (P = 0.033; **Figure 1F**), with expression levels progressively increasing with advancing stage. Our analysis also revealed a close association between PSMD14 expression and patient survival status. Among the top 25% of patients with the highest PSMD14 expression (Q1 group), the number of deaths was significantly higher than in other groups, suggesting that high PSMD14 expression may be linked to poorer prognosis in LUAD (**Figure 1G**). Consistently, Kaplan–Meier survival analysis demonstrated that patients with high PSMD14 expression had significantly worse overall survival (OS, p = 0.003), disease-specific survival (DSS, p = 0.001), progression-free interval (PFI, p < 0.001), and disease-free interval (DFI, p < 0.001) compared to those with low expression (**Figure 1H**). Moreover, both at the protein level (p < 0.001; **Figure 1I**) and in multiple GEO datasets of LUAD (Hazard Ratio > 1, p < 0.05; **Figure 1J**), low PSMD14 expression was associated with significantly better overall survival (p < 0.001). Collectively, these results indicate that PSMD14 expression may serve as a potential prognostic biomarker in LUAD and could help identify potential therapeutic targets.

### 3.2 PSMD14 is involved in multiple biological processes and signaling pathways in LUAD

**Figure 2:**
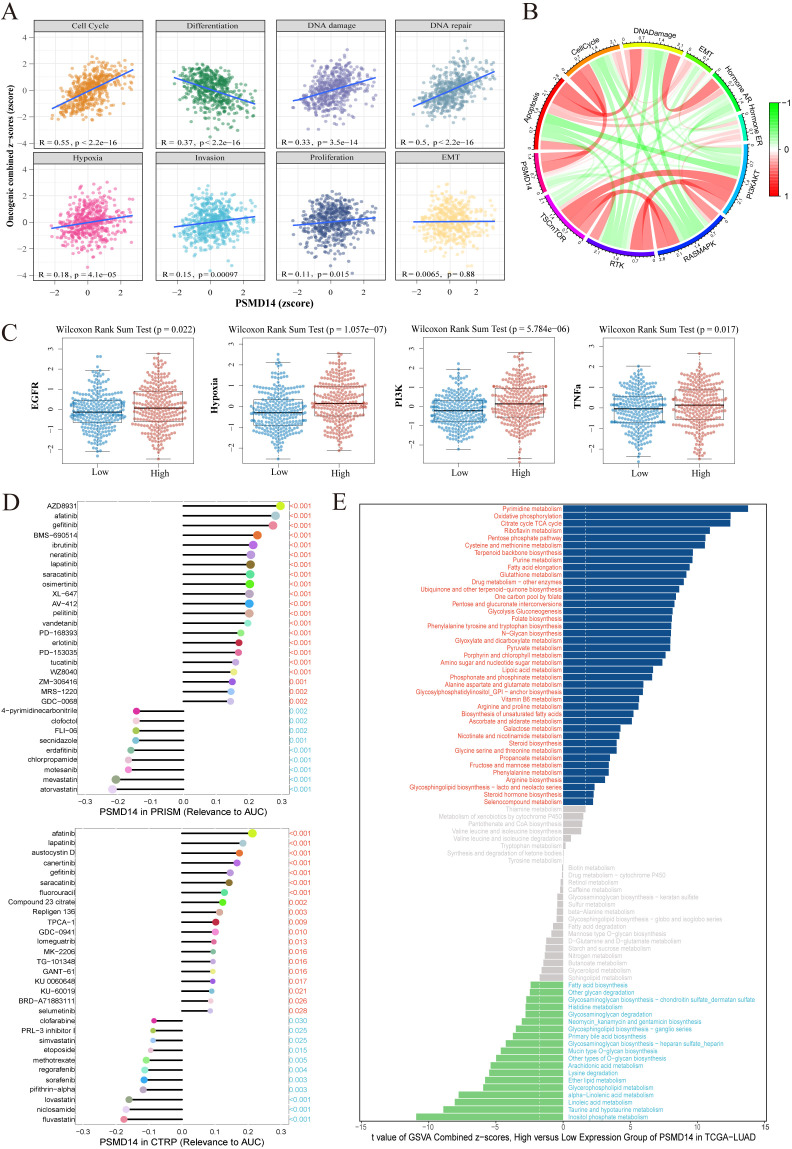
Correlation of PSMD14 expression with functional states, signaling pathways, drug sensitivity, and metabolic activity in LUAD. **(A)** Scatter plots showing the correlation between PSMD14 expression and activity scores of 8 functional states in LUAD. **(B)** Correlation of PSMD14 expression with pathway activity scores derived from TCPA-RPPA proteomic data. **(C)** Comparison of activity scores for multiple oncogenic pathways (EGFR, hypoxia, PI3K, TGF-β) between PSMD14 high- and low-expression groups in LUAD. **(D)** Correlation analysis between PSMD14 expression and sensitivity, represented by the area under the dose–response curve (AUC), to various common chemotherapeutic and targeted agents from the CTRP and PRISM databases. Higher AUC indicates lower drug sensitivity. **(E)** GSVA identifying specific metabolic pathways that are activated or suppressed in the PSMD14 high-expression group compared to the low-expression group in LUAD.

In LUAD, the expression of PSMD14 showed significant positive correlations with functional activity scores of cell cycle, DNA damage, and DNA repair. Specifically, Pearson correlation analysis revealed coefficients of 0.55 (p < 2.2e-16) for cell cycle, 0.33 (p < 3.5e-14) for DNA damage, and 0.5 (p < 2.2e-16) for DNA repair (**Figure 2A**), suggesting that PSMD14 expression is closely associated with the activity of these functional states and may play an important role in the pathogenesis of lung adenocarcinoma. Correlation analysis between gene expression and functional pathway activity quantified by TCPA-RPPA proteomic data revealed that PSMD14 expression positively correlates with apoptosis, cell cycle, epithelial-mesenchymal transition (EMT), and DNA damage response pathways in LUAD (**Figure 2B**). We also observed that pathway activity scores for EGFR signaling, hypoxia, PI3K, and TNF-a were significantly elevated in the PSMD14 high-expression group (**Figure 2C**), implying a statistically positive correlation between PSMD14 expression and the activation of these pathways.

Furthermore, leveraging the CTRP and PRISM databases, we assessed the correlation between PSMD14 expression and drug dose–response curves, as measured by the area under the curve (AUC). The analysis indicated that PSMD14 expression was positively correlated with AUC values of afatinib, gefitinib, erlotinib, and osimertinib—suggesting that higher PSMD14 expression is associated with reduced sensitivity to these EGFR-TKIs (**Figure 2D**).

GSVA further revealed that in LUAD, several metabolic pathways were enriched in the PSMD14 high-expression group, including pyrimidine metabolism, oxidative phosphorylation, and the citrate cycle (TCA cycle). In contrast, pathways such as inositol phosphate metabolism and glycerophospholipid metabolism were suppressed in the same group (**Figure 2E**).

### 3.3 The landscape of PSMD14 in the tumor microenvironment and its immunomodulatory role

**Figure 3:**
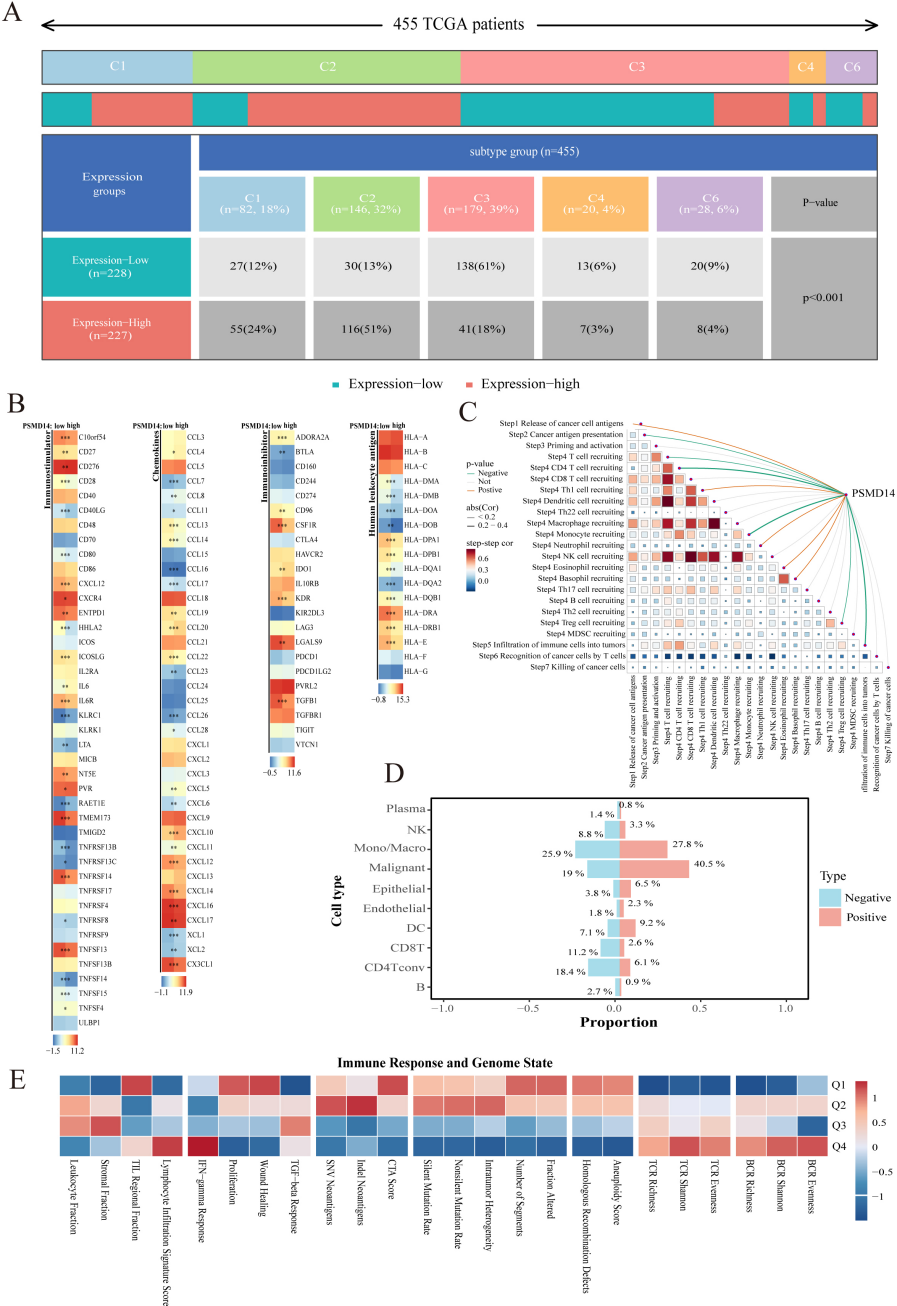
PSMD14 expression associates with immunosuppressive microenvironment in LUAD. **(A)** Distribution of six immune subtypes in PSMD14 high- versus low-expression groups (TCGA-LUAD cohort). **(B)** Expression patterns of immune-related molecules in PSMD14 high- and low-expression groups. Key upregulated molecules indicated. **(C)** Negative correlation between PSMD14 expression and Tumor Immunity Cycle (TIP) scores. **(D)** Single-cell RNA-seq reveals cellular composition: malignant cells enriched in PSMD14^+^ populations, CD8^+^ T cells in PSMD14^−^ populations. **(E)** PSMD14 expression correlates with distinct immune and genomic features: low expression with BCR/TCR diversity and IFN-γ response, high expression with DNA repair deficiency and proliferation. *P < 0.05, ** *P* < 0.01, ***P < 0.001.

Based on established literature (30), TCGA-LUAD samples were classified into six immune subtypes: C1 (wound healing), C2 (IFN-γ dominant), C3 (inflammatory), C4 (lymphocyte-depleted), C5 (immune silent), and C6 (TGF-β dominant). The C1 (24%) and C2 (51%) subtypes were enriched in the PSMD14 high-expression group, whereas the C3 subtype (61%) was predominant in the low-expression group. The C4 and C6 subtypes were underrepresented in both groups (**Figure 3A**), indicating distinct molecular features associated with PSMD14 expression. A heatmap visualized the expression of immune-related molecules—including immunomodulators, chemokines, and HLA genes—between PSMD14 high- and low-expression groups. Key molecules such as CD276, CD80, CXCL5, and CXCL10 were upregulated in the PSMD14 high-expression group (**Figure 3B**).

Spearman correlation analysis revealed a significant negative correlation between PSMD14 expression and Tumor Immunity Cycle (TIP) (31), scores in LUAD (**Figure 3C**), suggesting that elevated PSMD14 levels may impair anti-tumor immune activity. Cell subset analysis showed that malignant cells were significantly enriched in the PSMD14-positive group (40.5% vs. 19% in the negative group), suggesting their major contribution to PSMD14 expression. In contrast, CD8^+^ T cells were more abundant in the PSMD14-negative group (11.2% vs. 2.6%) (**Figure 3D**). Patients were stratified into Q1–Q4 quartiles by PSMD14 expression. Low PSMD14 expression (Q4) correlated with BCR Shannon diversity, TCR Shannon diversity, and IFN-γ response, while high expression (Q1) was associated with Homologous Recombination Deficiency, Wound Healing, and Proliferation signatures (**Figure 3E**).

Collectively, these findings indicate that elevated PSMD14 expression is associated with an immunosuppressive and inflammatory tumor microenvironment (TME) in LUAD. This specific TME facilitates tumor immune evasion, thereby promoting tumor growth and malignant progression.

### 3.4 HMMR is positively regulated by PSMD14

**Figure 4:**
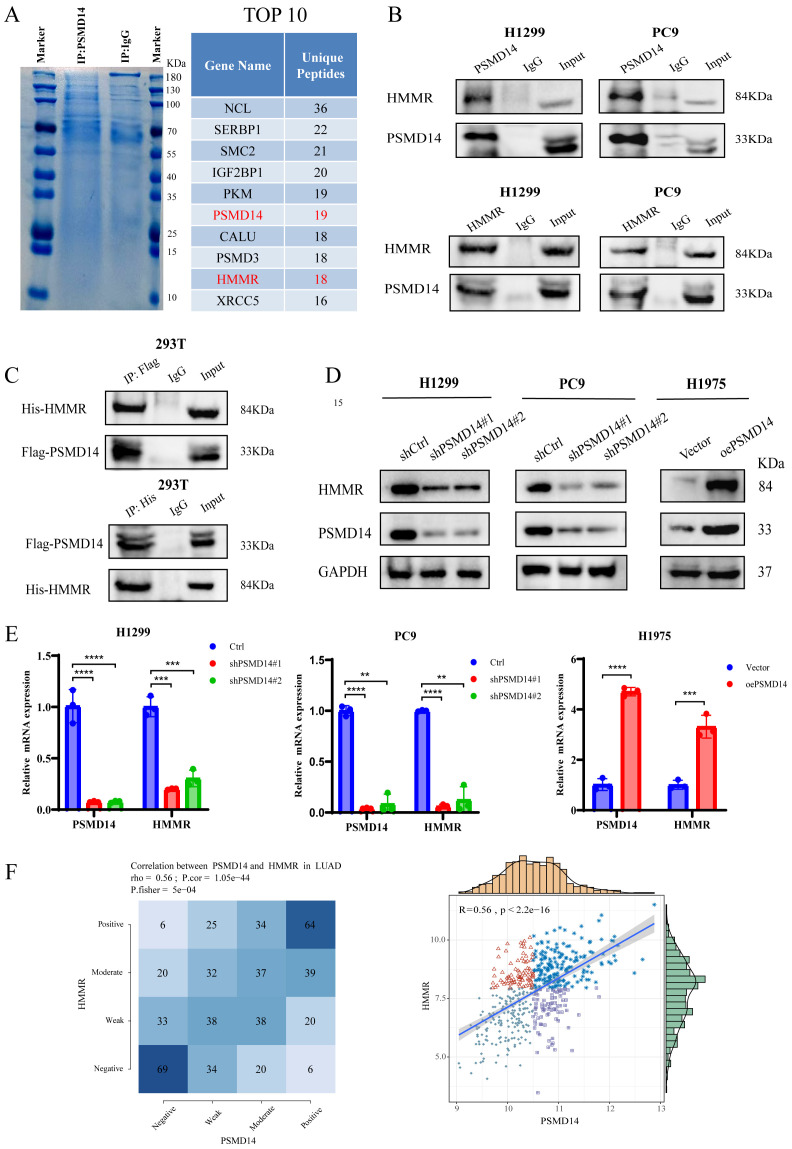
PSMD14 interacts with and positively regulates HMMR expression. **(A)** IP-MS analysis identifies potential PSMD14-interacting proteins in H1299 cells overexpressing PSMD14. **(B, C)** Co-immunoprecipitation assays confirm the interaction between PSMD14 and HMMR in LUAD cells. **(D, E)** Knockdown and overexpression of PSMD14 demonstrate its positive regulation of HMMR expression at both mRNA and protein levels. **(F)** Analysis of TCGA-LUAD data reveals significant positive correlation between PSMD14 and HMMR mRNA expression in clinical samples. **P < 0.01, ***P < 0.001, ****P < 0.0001.

To further elucidate the molecular mechanisms by which PSMD14 promotes LUAD progression, we performed immunoprecipitation combined with mass spectrometry (IP-MS) in H1299 cells overexpressing PSMD14, which identified multiple potential binding partners of PSMD14 (**Figure 4A**). To screen for key downstream molecules of PSMD14, we conducted an initial bioinformatic analysis using the GEPIA database. Among the most abundant proteins in the PSMD14 interactome identified by IP-MS, HMMR was significantly upregulated in LUAD tumors compared with adjacent normal tissues and correlated with poor patient prognosis (**Supplementary Figures S1A, B**). In contrast, NCL showed no significant association with either expression or prognosis, while SERBP1, SMC2, IGF2BP1, and PKM—though not markedly overexpressed in LUAD—were also linked to unfavorable survival outcomes (**Supplementary Figures S1A, B**). Subsequently, correlation analysis revealed positive associations between PSMD14 expression and that of HMMR, SMC2, PKM, IGF2BP1, and SERBP1, with Pearson correlation coefficients of 0.53, 0.42, 0.4, 0.37, and 0.34, respectively (**Supplementary Figure S1C**). Finally, experimental validation showed that only HMMR exhibited a pronounced change in protein expression upon PSMD14 overexpression in H1975 cells (**Supplementary Figure S1D**), leading us to select HMMR for further investigation. Using co-immunoprecipitation (Co-IP) assays, we confirmed a specific interaction between PSMD14 and HMMR (**Figures 4B, C**). Subsequent knockdown and overexpression of PSMD14 in LUAD cell lines demonstrated that HMMR expression is positively regulated by PSMD14 at both the mRNA and protein levels (**Figures 4D, E**). These results establish HMMR as a direct interacting partner of PSMD14.

**Figure 5:**
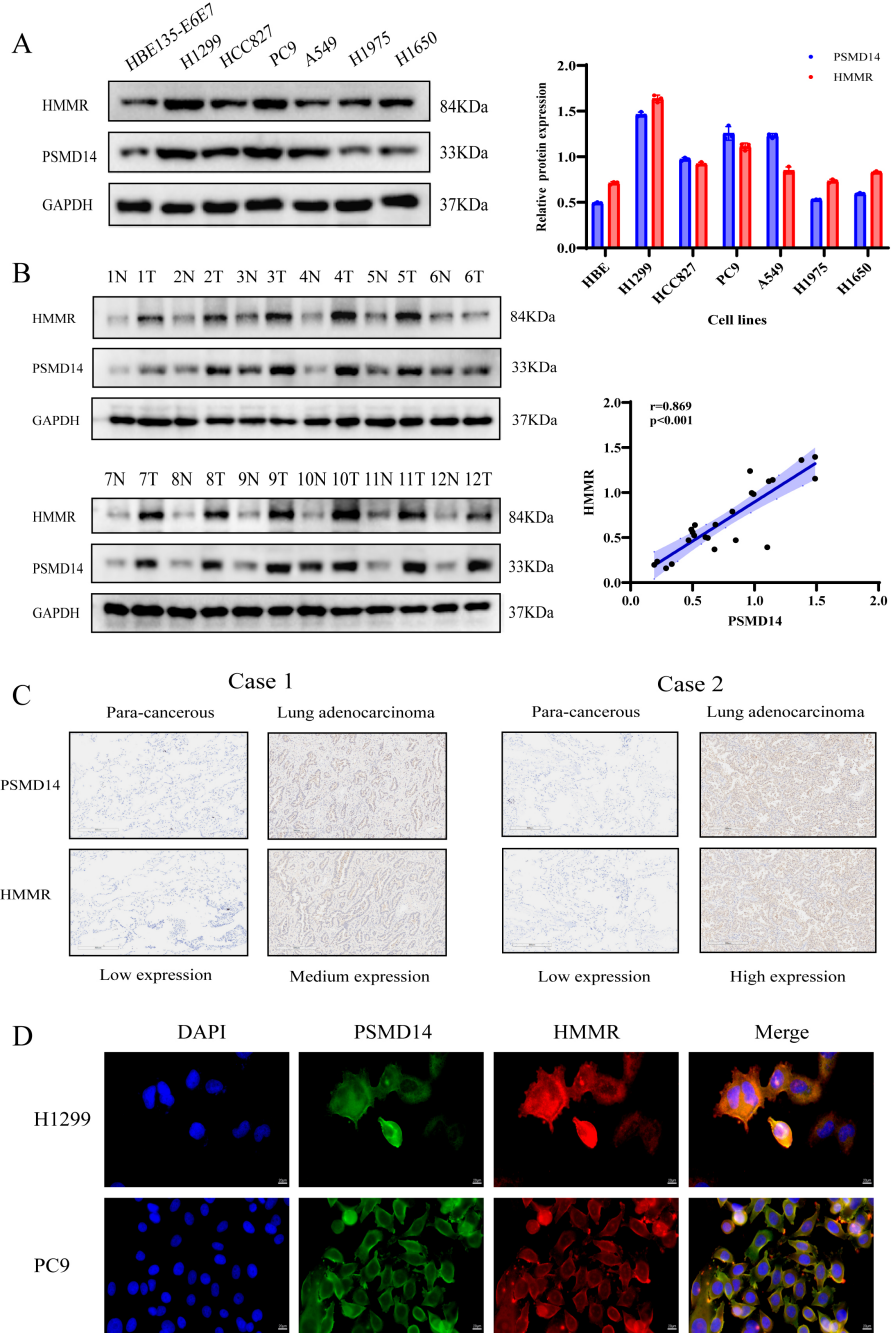
Coordinated upregulation of PSMD14 and HMMR in LUAD tissues. **(A)** Western blot showing elevated protein expression of both PSMD14 and HMMR in multiple LUAD cell lines compared to normal bronchial epithelial cells. **(B)** Western blot and scatter plot demonstrating strong positive correlation between PSMD14 and HMMR protein levels in 12 paired LUAD and adjacent normal tissues. **(C)** IHC staining showing concurrent upregulation of PSMD14 and HMMR proteins in LUAD specimens compared to adjacent normal tissues. **(D)** Immunofluorescence imaging revealing spatial co-localization of PSMD14 and HMMR in LUAD cells.

Analysis of TCGA-LUAD data revealed a significant positive correlation between PSMD14 and HMMR expression in clinical specimens (r = 0.56, p < 0.001; **Figure 4F**). At the protein level, both PSMD14 and HMMR were markedly upregulated in multiple LUAD cell lines compared to normal bronchial epithelial cells (**Figure 5A**). A significant positive correlation between their expression levels was also confirmed across these cell lines (r = 0.804, p = 0.029; **Supplementary Figure S1E**). Furthermore, this correlation was validated in clinical specimens. Analysis of 12 paired LUAD and adjacent normal tissues from The Second Affiliated Hospital of Nanchang University revealed a strong positive correlation between PSMD14 and HMMR protein levels (r = 0.869, p < 0.001; **Figure 5B**), where HMMR expression was significantly elevated in PSMD14-high tissues. Moreover, IHC analysis of LUAD specimens from the same source further confirmed the coordinated upregulation of both PSMD14 and HMMR in tumor tissues compared to adjacent normal regions (**Figure 5C**). Immunofluorescence staining further revealed their spatial co-localization in LUAD cells (**Figure 5D**). Collectively, these findings demonstrate a positive regulatory relationship between PSMD14 and HMMR in LUAD.

### 3.5 HMMR as a direct target gene of PSMD14

**Figure 6:**
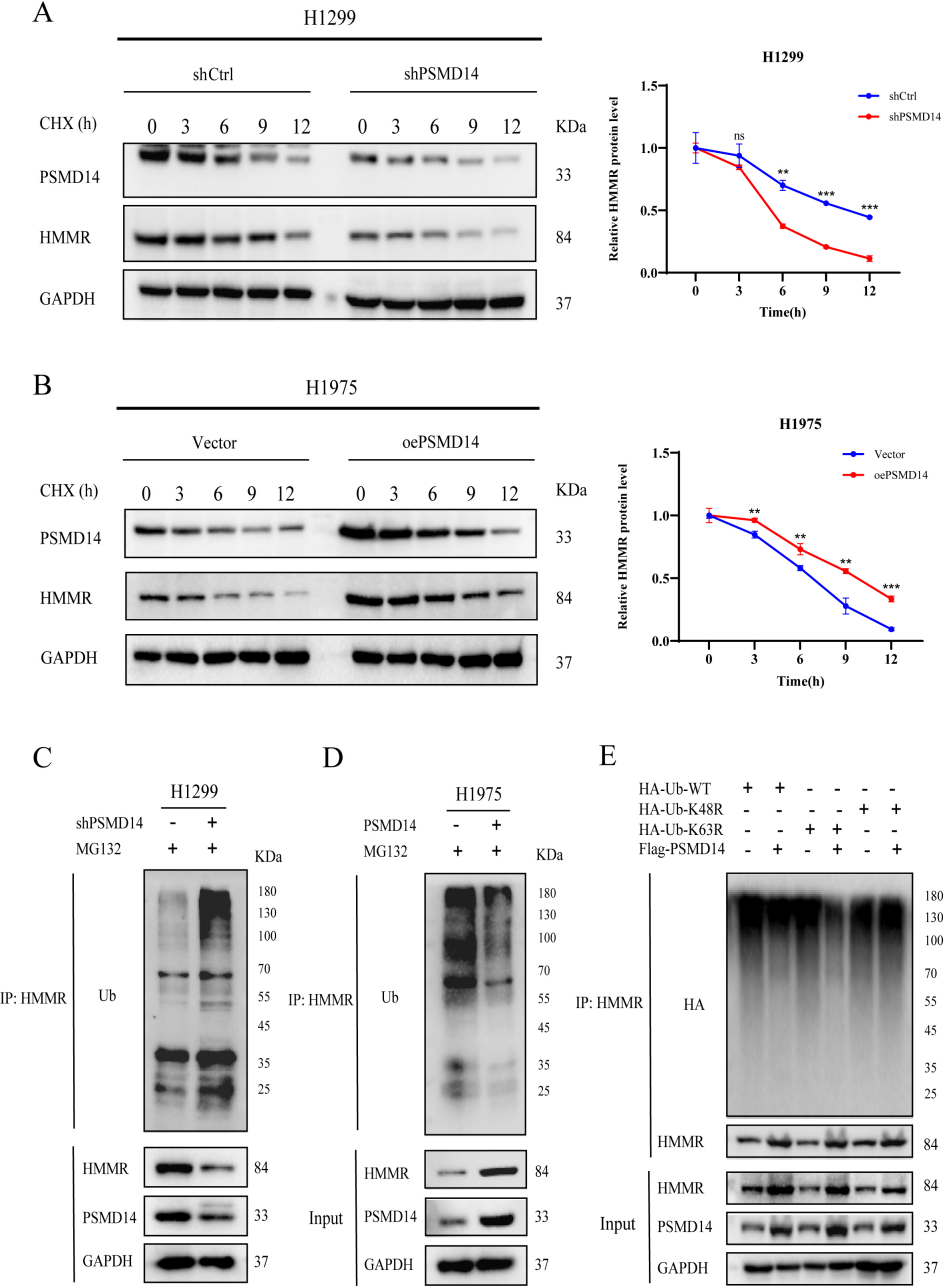
PSMD14 stabilizes HMMR through K63-linked deubiquitination. **(A, B)** Cycloheximide (CHX) chase assays show that PSMD14 knockdown reduces while its overexpression extends the half-life of HMMR protein in LUAD cells. **(C, D)** Ubiquitination assays demonstrate that PSMD14 knockdown enhances whereas its overexpression reduces ubiquitination of HMMR in MG132-treated cells. **(E)** Ubiquitination analysis using K48R and K63R mutants reveals that PSMD14 specifically removes K63-linked ubiquitin chains from HMMR. ‘ns’ indicates non-significance, ** *P* < 0.01, ***P < 0.001.

Given the established role of PSMD14 as a deubiquitinating enzyme in multiple cancers, we sought to investigate whether it regulates HMMR protein stability. We first treated PSMD14-knockdown H1299 cells with cycloheximide (CHX) and observed a shortened half-life of HMMR (**Figure 6A**). Conversely, PSMD14 overexpression in H1975 cells extended the half-life of HMMR (**Figure 6B**). To determine whether this stabilization depends on the deubiquitinating activity of PSMD14, we measured HMMR ubiquitination levels in MG132-treated LUAD cells upon PSMD14 knockdown or overexpression. As shown in **Figures 6C, D**, PSMD14 knockdown markedly enhanced HMMR ubiquitination, whereas its overexpression reduced it. To further characterize the linkage specificity, we examined HMMR ubiquitination following PSMD14 overexpression using antibodies specific to K63- and K48-linked ubiquitin chains. These results indicated that PSMD14 preferentially removes K63-linked ubiquitin chains from HMMR (**Supplementary Figure S1F**). To corroborate this finding, we performed ubiquitination assays using ubiquitin mutants K48R and K63R, in which lysine residues at position 48 or 63 are replaced by arginine. Consistent with the previous observation, PSMD14 primarily cleaved K63-linked ubiquitin chains from HMMR (**Figure 6E**). Taken together, these data demonstrate that PSMD14 stabilizes HMMR through a deubiquitination mechanism that predominantly targets K63-linked ubiquitin chains.

### 3.6 PSMD14 promotes LUAD cell proliferation and regulates cell cycle

**Figure 7:**
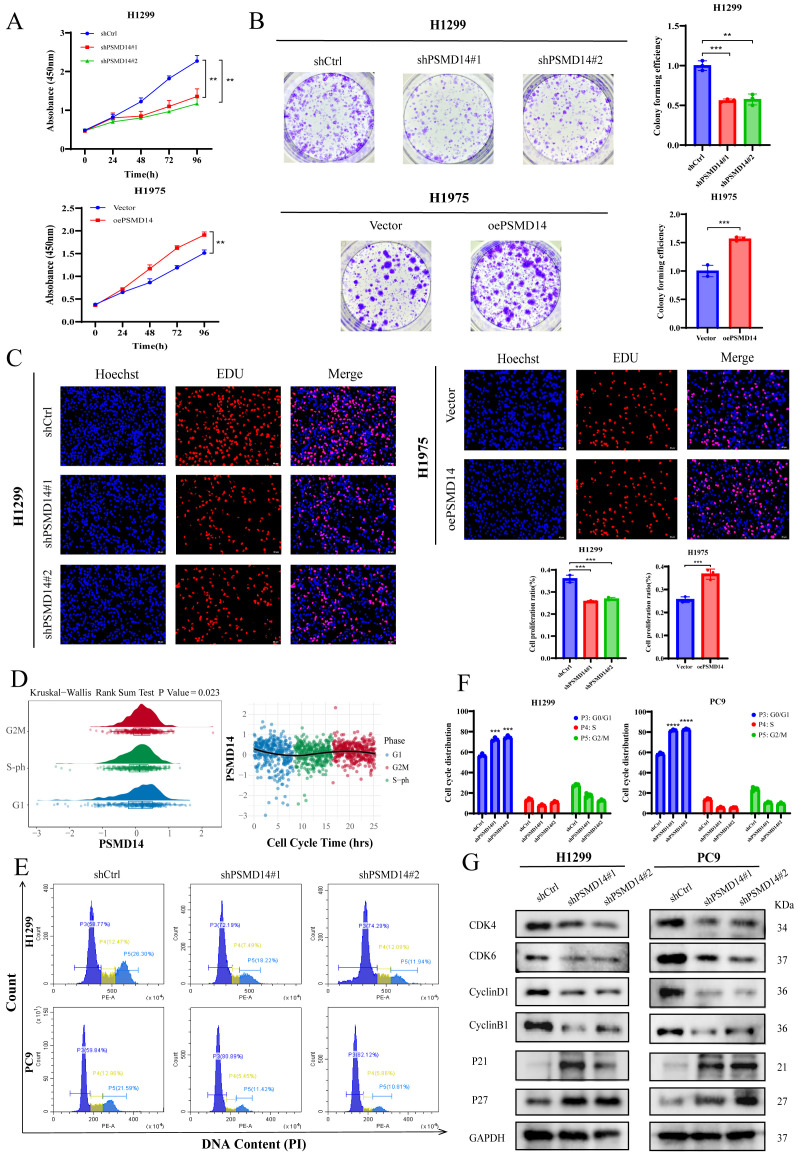
PSMD14 promotes proliferation of lung adenocarcinoma cells and regulates cell cycle progression. **(A)** CCK-8 assay shows that PSMD14 knockdown inhibits proliferation of H1299, while its overexpression enhances proliferation of H1975 cells. **(B)** Colony formation assay demonstrates that PSMD14 knockdown reduces colony-forming ability in H1299, whereas its overexpression increases colony formation in H1975 cells. **(C)** EdU assay further confirms the pro-proliferative effect of PSMD14 in LUAD cells. ** *P* < 0.01, ***P < 0.001, ****P < 0.0001. **(D)** Single-cell RNA sequencing analysis depicting PSMD14 expression dynamics across cell cycle phases. **(E)** Flow cytometric analysis of cell cycle distribution following PSMD14 knockdown in LUAD cells. **(F)** Depletion of PSMD14 induces G0/G1 phase arrest in LUAD cells. **(G)** Western blot showing expression changes of key cell cycle regulators upon PSMD14 knockdown.

To further investigate the biological function of PSMD14 in LUAD progression, we established PSMD14-knockdown models in H1299 and PC9 cells and a PSMD14-overexpression model in H1975 cells. CCK-8 assays revealed that PSMD14 knockdown significantly suppressed proliferation in H1299 and PC9 cells, while its overexpression markedly enhanced proliferation in H1975 cells (**Figure 7A**, **Supplementary Figure S2A**). Colony formation assays yielded consistent results (**Figure 7B**, **Supplementary Figure S2B**). Furthermore, EdU assays confirmed the inhibitory effect of PSMD14 knockdown and the promotive effect of its overexpression on cellular proliferation (**Figure 7C**, **Supplementary Figure S2C**). Collectively, these findings demonstrate that PSMD14 promotes the proliferation of LUAD cells.

Based on single-cell RNA sequencing data, we determined RNA expression levels and cell cycle phases at single-cell resolution. By plotting normalized RNA expression along a linear pseudotime trajectory defined by cell cycle marker intensity, we observed distinct expression patterns of PSMD14 across different cell cycle phases, revealing its dynamic regulation during cell cycle progression (**Figure 7D**). We subsequently performed flow cytometry to analyze cell cycle alterations following PSMD14 knockdown in H1299 and PC9 cells. The results demonstrated a significant G1 phase arrest (**Figures 7E, F**), which was consistent with subsequent Western blot analysis showing decreased expression of CDK4, CDK6, Cyclin B1, and Cyclin D1, along with increased levels of P21 and P27 (**Figure 7G**).

### 3.7 PSMD14 promotes migration, invasion, and regulates DNA damage repair in LUAD Cells

**Figure 8:**
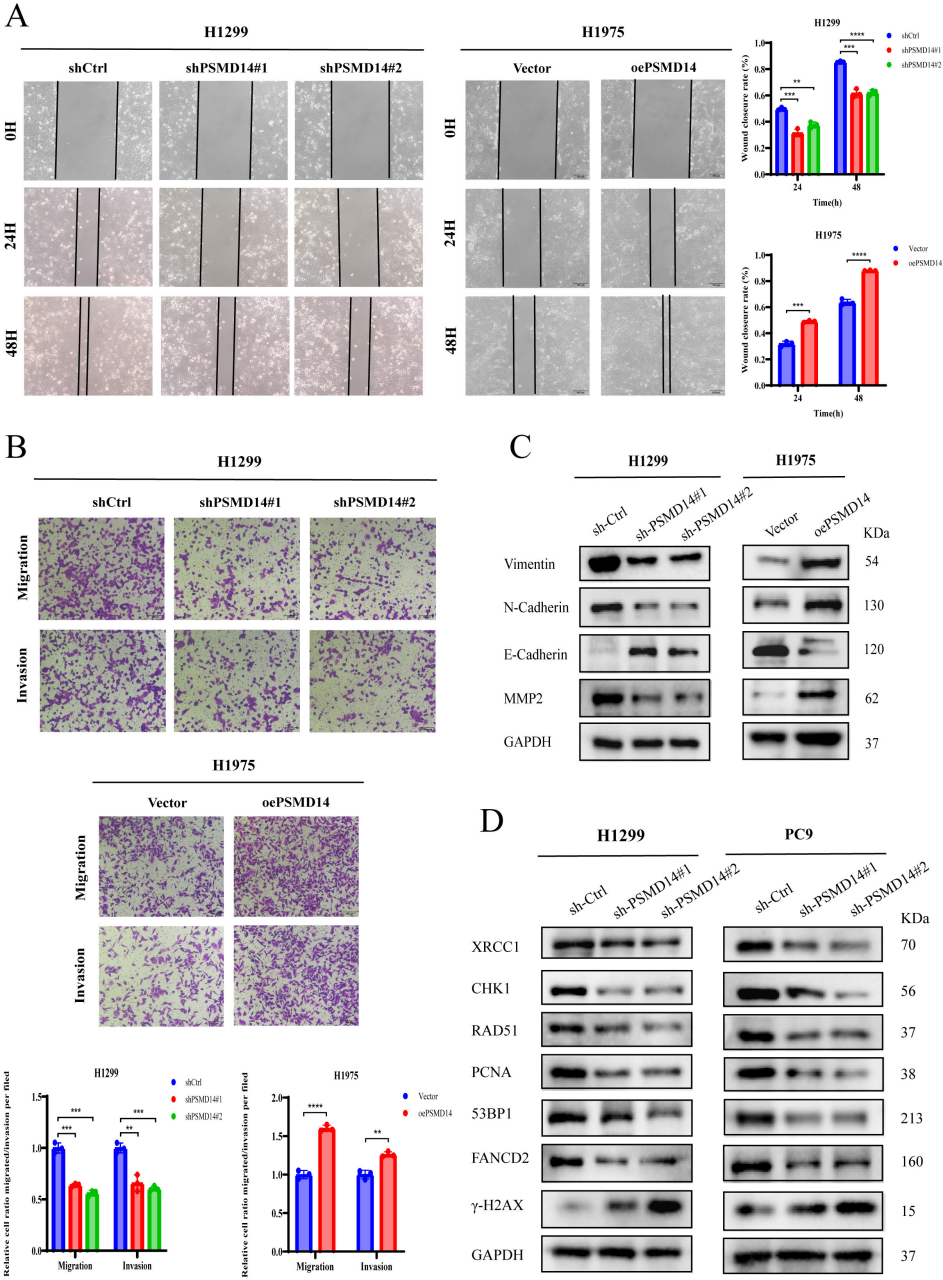
PSMD14 enhances migration and invasion capabilities of LUAD cells and regulates DNA damage repair in LUAD. **(A)** Wound healing assay reveals that PSMD14 knockdown impairs cell migration in LUAD cells, while its overexpression promotes migratory capacity. **(B)** Transwell assay indicates that PSMD14 silencing attenuates migration and invasion abilities of LUAD cells, whereas its overexpression enhances these malignant phenotypes. **(C)** Western blot analysis of epithelial-mesenchymal transition (EMT)-related markers after PSMD14 knockdown. **(D)** Western blot analysis of DNA damage repair proteins following PSMD14 depletion. **P < 0.01, ***P < 0.001, ****P < 0.0001.

In wound healing assays, PSMD14-knockdown LUAD cells exhibited impaired migration capability compared to shCtrl-transfected controls, whereas PSMD14 overexpression produced the opposite effect in H1975 cells (**Figure 8A**, **Supplementary Figure S2D**). Similar results were obtained from Transwell assays, which showed that PSMD14 silencing significantly attenuated both migration and invasion in H1299 and PC9 cells, while PSMD14 overexpression enhanced these capabilities in H1975 cells (**Figure 8B**, **Supplementary Figure S2E**). Together, these data indicate that PSMD14 significantly promotes the migratory and invasive abilities of LUAD cells *in vitro*. Furthermore, we examined the correlation between PSMD14 and the expression of epithelial-mesenchymal transition (EMT)-related as well as DNA damage repair-associated proteins by Western blot. PSMD14 knockdown led to reduced expression of Vimentin, N-Cadherin, and MMP2, while E-Cadherin was upregulated (**Figure 8C**).

Our analysis revealed that PSMD14 expression positively correlates with multiple genomic instability indices, including aneuploidy, homologous recombination deficiency (HRD), nonsilent and silent mutation rates, and SNV neoantigen load. The positive association with HRD scores suggests that tumors with elevated PSMD14 expression may exhibit increased DNA repair defects, potentially rendering them more sensitive to certain chemotherapeutic agents (e.g., platinum-based drugs) and PARP inhibitors. Elevated nonsilent mutation rates may lead to alterations in protein function, structure, or stability, thereby influencing cellular physiology and phenotype. SNV neoantigens—aberrant proteins resulting from single nucleotide variations that can be recognized by the immune system—represent critical targets for cancer immunotherapy. Our Western blot analysis further demonstrated that knockdown of PSMD14 in LUAD cells led to a decrease in the protein levels of XRCC1, RAD51, 53BP1, and FANCD2, while increasing the level of γ-H2AX (**Figure 8D**). Overall, the positive correlations between PSMD14 expression and these genomic instability metrics indicate that high PSMD14 expression is associated with increased chromosomal instability in LUAD (**Supplementary Figure S2F**).

### 3.8 HMMR is required for PSMD14-mediated malignant progression in LUAD

**Figure 9:**
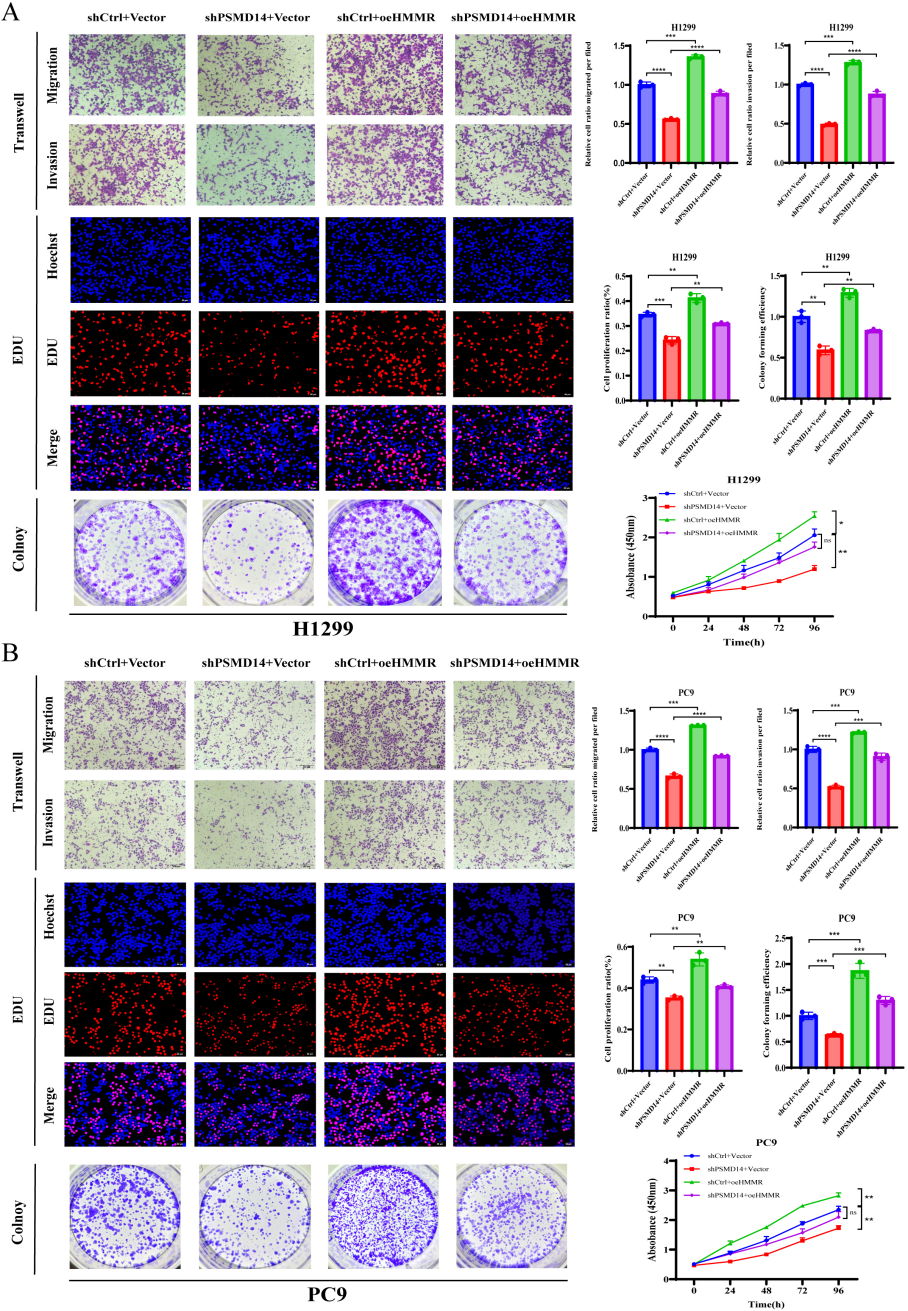
PSMD14 promotes proliferation, migration, and invasion of LUAD cells by targeting HMMR. **(A)** HMMR overexpression rescues PSMD14-knockdown phenotypes in H1299 cells (CCK-8, colony formation, and Transwell assays). **(B)** Similar rescue of malignant phenotypes by HMMR in PSMD14-deficient PC9 cells. *P < 0.05, **P < 0.01, ***P < 0.001, ****P < 0.0001.

Based on the functional roles of PSMD14 in LUAD pathogenesis and HMMR expression regulation, we hypothesized that PSMD14 promotes LUAD progression partially through HMMR. To test this hypothesis, we overexpressed HMMR in PSMD14-knockdown H1299 and PC9 cells and compared their malignant phenotypes with those exhibiting PSMD14 knockdown or HMMR overexpression alone. Functional assays demonstrated that HMMR overexpression substantially rescued the impaired proliferation, migration, and invasion capabilities resulting from PSMD14 knockdown (**Figures 9A, B**). Taken together, these results indicate that PSMD14 accelerates LUAD progression by targeting HMMR.

### 3.9 Capzimin suppresses proliferation and migration while inducing apoptosis in LUAD

**Figure 10:**
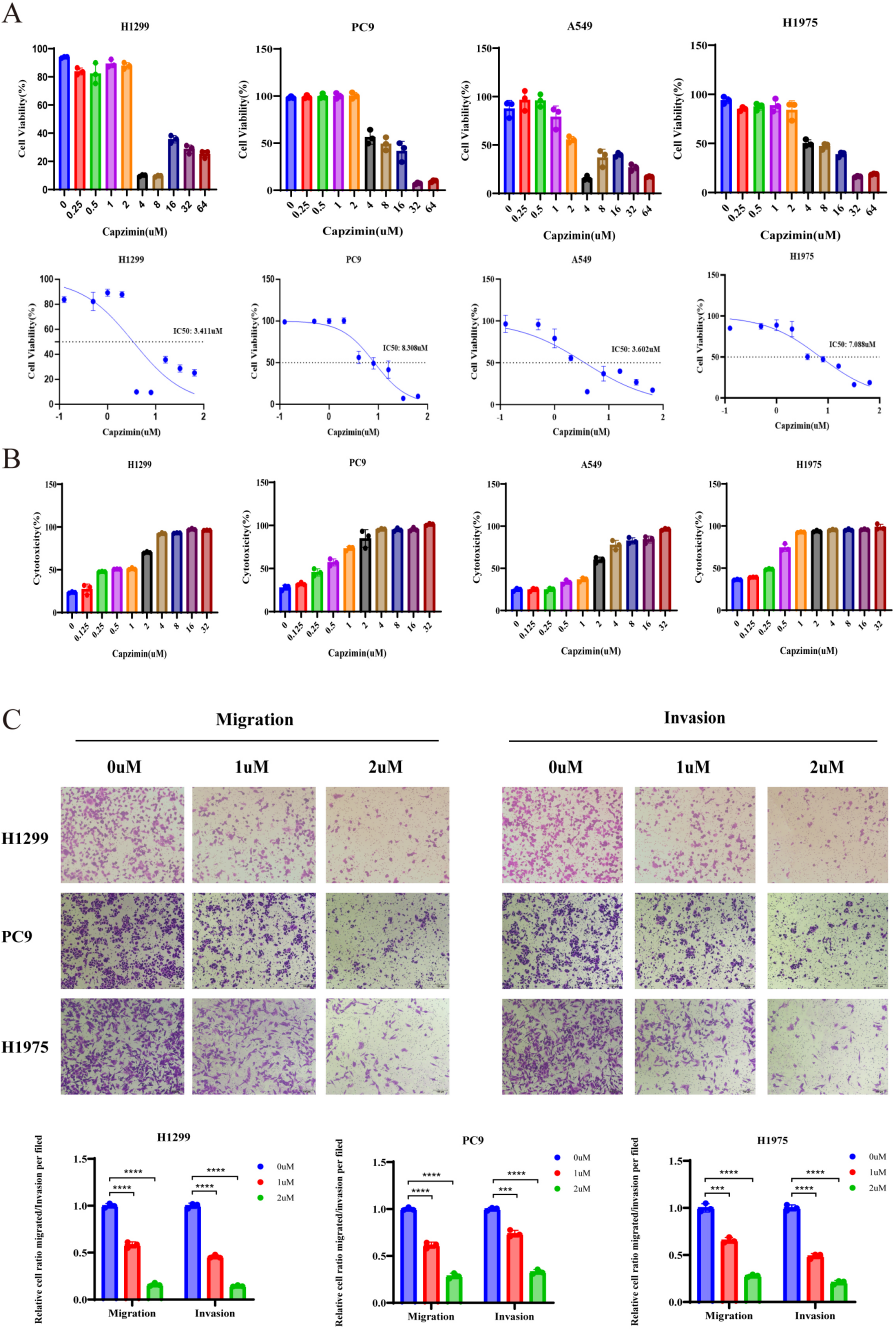
Capzimin exhibits cytotoxic effects and inhibits migration in LUAD cells. **(A)** IC_50_ values of Capzimin in indicated LUAD cell lines. **(B)** Cytotoxicity assessment of Capzimin at low concentrations in LUAD cells. **(C)** Transwell assays demonstrated that treatment with Capzimin reduced the migratory and invasive capacities of LUAD cells. ***P < 0.001, ****P < 0.0001.

**Figure 11:**
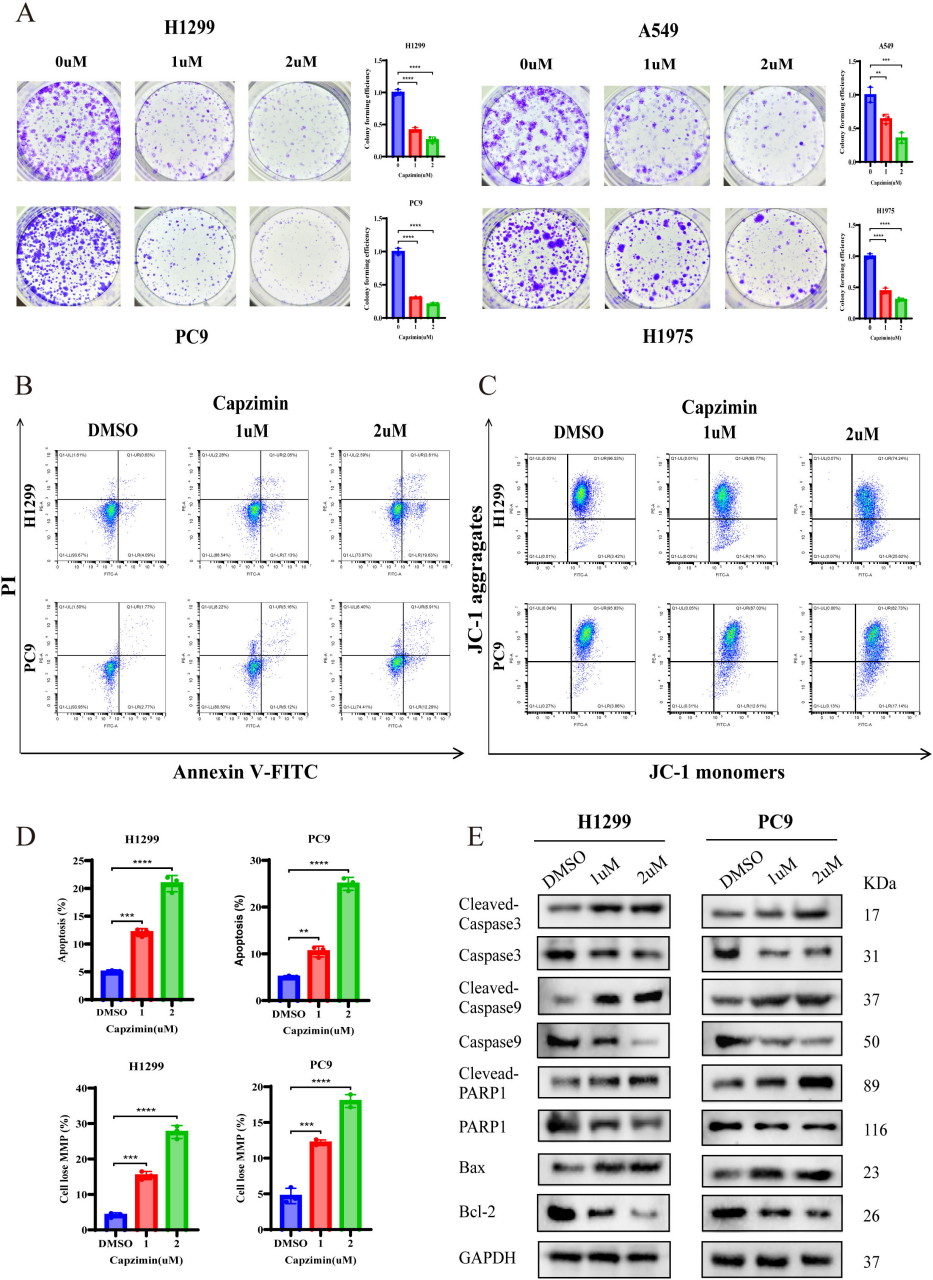
Capzimin suppresses colony formation and induces mitochondrial-mediated apoptosis in LUAD cells. **(A)** Colony formation assays of LUAD cells after Capzimin treatment. **(B, C)** Flow cytometric analysis of apoptosis **(B)** and mitochondrial membrane potential **(C)** in Capzimin-treated LUAD cells. **(D)** Quantitative analysis of apoptosis rate and mitochondrial membrane potential changes in LUAD cells following Capzimin treatment. **(E)** Western blot analysis of apoptosis-related proteins in LUAD cells following Capzimin treatment. **P < 0.01, ***P < 0.001, ****P < 0.0001.

Capzimin has been identified as a potent inhibitor of PSMD14 (32). We first determined the IC_50_ values of Capzimin in multiple LUAD cell lines, which were 3.411 µM in H1299, 8.308 µM in A549, 3.602 µM in PC9, and 7.088 µM in H1975 cells (**Figure 10A**). Subsequent cytotoxicity assays revealed that even low concentrations of Capzimin significantly reduced LUAD cell viability (**Figure 10B**). Furthermore, Capzimin treatment effectively suppressed the proliferative and metastatic capacities of LUAD cells. Specifically, Transwell assays demonstrated a marked reduction in the number of migrating and invading cells following Capzimin exposure in H1299, A549, PC9, and H1975 lines (**Figure 10C**). Additionally, colony formation was significantly impaired in Capzimin-treated LUAD cells (**Figure 11A**). More importantly, Capzimin notably induced apoptosis and loss of mitochondrial membrane potential (**Figures 11B–D**). Consistent with these observations, Western blot analysis showed upregulation of cleaved caspase-3, cleaved caspase-9, cleaved PARP-1, and Bax, along with downregulation of Bcl-2 in drug-treated cells (**Figure 11E**).

### 3.10 PSMD14 promotes LUAD progression via TGF-β/Smad and PI3K/AKT/mTOR signaling pathways

**Figure 12:**
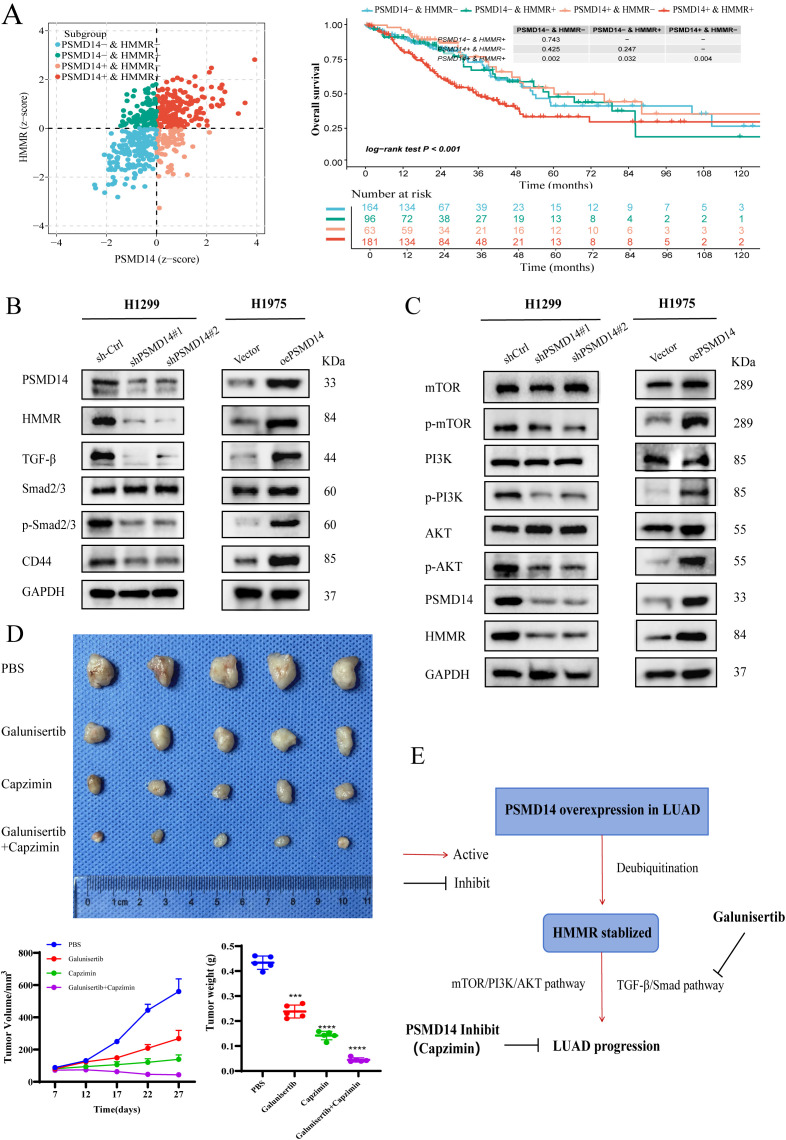
PSMD14 promotes LUAD progression through HMMR-mediated activation of TGF-β/Smad and PI3K/AKT/mTOR signaling pathways. **(A)** Kaplan-Meier survival analysis of LUAD patients stratified by PSMD14 and HMMR co-expression patterns. **(B)** Western blot analysis of TGF-β/Smad pathway components and CD44 expression following PSMD14 modulation. **(C)** Western blot analysis of PI3K/AKT/mTOR pathway components after PSMD14 knockdown or overexpression. **(D)** Tumor growth curves in PC9 xenograft models treated with Capzimin, Galunisertib, or their combination. **(E)** Schematic diagram of the potential mechanism of PSMD14 in LUAD progression. ***P < 0.001, ****P < 0.0001.

Based on gene expression patterns, TCGA-LUAD samples were stratified into four distinct subgroups: PSMD14^+^HMMR^+^, PSMD14^−^HMMR^+^, PSMD14^−^HMMR^−^, and PSMD14^+^HMMR^−^. Kaplan-Meier survival analysis revealed that patients in the PSMD14^+^HMMR^+^ subgroup had significantly poorer overall survival compared to the PSMD14^−^HMMR^−^ subgroup (log-rank test, p < 0.001). Notably, no significant survival differences were observed between the PSMD14^−^HMMR^−^ subgroup and either the PSMD14^−^HMMR^+^ or PSMD14^+^HMMR^−^ subgroups. However, the PSMD14^+^HMMR^+^ subgroup showed significantly worse survival compared to both the PSMD14^+^HMMR^−^ and PSMD14^−^HMMR^+^ subgroups (**Figure 12A**). These findings indicate that high expression of either PSMD14 or HMMR alone does not substantially impact patient prognosis, whereas their concurrent overexpression leads to significantly worse outcomes, suggesting a synergistic effect between PSMD14 and HMMR in promoting LUAD progression.

To elucidate the mechanisms underlying PSMD14-mediated LUAD progression, we examined its effects on key signaling pathways. Compared to controls, PSMD14 knockdown reduced TGF-β, p-Smad2/3 phosphorylation, and CD44 expression, whereas PSMD14 overexpression increased these markers without altering total protein levels (**Figure 12B**). Similarly, PSMD14 knockdown decreased phosphorylation of mTOR, PI3K, and AKT, while its overexpression enhanced their activation, with no changes in total protein expression (**Figure 12C**). These results indicate that PSMD14 regulates both TGF-β/Smad and PI3K/AKT/mTOR signaling pathways.

In xenograft models established by injecting PC9 cells, the combination of Capzimin and galunisertib demonstrated synergistic suppression of tumor growth, yielding superior antitumor efficacy compared to either agent alone (**Figure 12D**). Collectively, these findings demonstrate that PSMD14 stabilizes HMMR protein through deubiquitination and promotes LUAD progression by activating TGF-β/Smad and PI3K/AKT/mTOR signaling pathways (**Figure 12E**).

## 4 Discussion

LUAD continues to pose a significant clinical challenge as a leading cause of cancer-related mortality worldwide, with particularly limited therapeutic options and poor five-year survival rates for patients with advanced-stage disease (1). This pressing clinical reality underscores the urgent need to identify novel prognostic biomarkers and therapeutic targets. Protein ubiquitination, an essential post-translational modification, represents a multi-step enzymatic cascade that governs diverse cellular processes including protein degradation, signal transduction, and DNA repair (33). The delicate balance of ubiquitination is precisely regulated by the opposing actions of ubiquitin-conjugating enzymes and deubiquitinating enzymes (DUBs). Dysregulation of this equilibrium has been mechanistically linked to tumor initiation and progression across various cancer types (9).

Notably, DUB-mediated deubiquitination has emerged as a crucial regulatory mechanism in NSCLC pathogenesis, with accumulating evidence supporting its roles in modulating key oncogenic pathways and therapeutic resistance. For instance, USP7 promotes non-small cell lung cancer progression by deubiquitinating and stabilizing KRAS (34). USP9X-mediated deubiquitination of KDM4C epigenetically induces TGF-β2 transcription to drive radioresistance in lung cancer (35). Additionally, USP28-mediated FOXK1 deubiquitination activates the Hippo signaling pathway to regulate cell proliferation and radiosensitivity (36), while targeting USP47 enhances the efficacy of KRAS G12C inhibitors in mutant non-small cell lung cancer through regulation of c-Myc deubiquitination (37). These compelling examples not only validate DUBs as promising therapeutic targets but also provide strong rationale for further exploration of the DUB family in LUAD pathogenesis and treatment.

Accumulating evidence has established the deubiquitinase PSMD14 as a critical promoter of tumor progression across diverse cancers, primarily through a conserved mechanism of stabilizing oncogenic substrates via K63-linked deubiquitination. For instance, in breast cancer, PSMD14 removes K63-linked ubiquitin chains from FOXM1 to activate the PI3K/AKT/mTOR pathway, thereby facilitating malignant progression (13). Similarly, in hepatocellular carcinoma, PSMD14-mediated deubiquitination stabilizes CARM1, leading to the transcriptional activation of FERMT1 and subsequent promotion of proliferation and metastasis (17). Notably, a parallel mechanism was reported in lung cancer, where PSMD14 promotes metastasis by deubiquitinating Smad3 to augment TGF-β1/Smad3 signaling and is associated with poor patient prognosis (21, 38). These collective findings underscore the pivotal and multi-faceted oncogenic roles of PSMD14. While these studies establish a paradigm of PSMD14 activating individual oncogenic pathways in a context-dependent manner, its functional repertoire in LUAD, particularly the ability to co-regulate multiple core pathways and influence the tumor immune landscape, remained largely unexplored. This gap in knowledge highlights the need for the present study, which aims to systematically investigate the role of PSMD14 in LUAD pathogenesis.

In the study, the clinical significance of PSMD14 is underscored by its robust performance as both a diagnostic and prognostic biomarker in LUAD. With an AUC of 0.898, PSMD14 demonstrates exceptional diagnostic accuracy that surpasses many previously reported biomarkers in LUAD. More importantly, its prognostic value extends across multiple survival metrics including overall survival, disease-specific survival, progression-free interval, and disease-free interval. This consistent prognostic performance across independent datasets and at the protein level strengthens its potential clinical utility. Interestingly, our immune profiling revealed that PSMD14 high expression associates with an immunosuppressive microenvironment characterized by specific immune subtypes and reduced CD8+ T cell infiltration, suggesting its potential role in modulating anti-tumor immunity. These results are consistent with a recent study highlighting PSMD14’s role in the pervasive immune evasion mechanisms of LUAD (12).

At the molecular level, we made the crucial discovery that PSMD14 directly interacts with and stabilizes HMMR through removal of K63-linked ubiquitin chains. This finding is particularly significant as HMMR has emerged as a key player in cancer progression through its roles in regulating cell cycle progression and genomic stability (39). More importantly, our study reveals that the PSMD14-HMMR axis represents a distinct and integrative oncogenic mechanism in LUAD. Unlike previously reported roles of PSMD14 in activating either the TGF-β pathway or the PI3K pathway in isolation, our work is the first to demonstrate that PSMD14 can coordinately activate both of these core signaling pathways in LUAD, thereby driving more aggressive malignant phenotypes. This finding provides a mechanistic basis for the potent oncogenicity of PSMD14-HMMR axis. Furthermore, our novel exploration links the PSMD14-HMMR axis to the establishment of an immunosuppressive tumor microenvironment, an aspect of PSMD14 biology not previously associated with its canonical deubiquitination function. The PSMD14-HMMR axis thus represents a novel regulatory mechanism in LUAD, potentially explaining the observed correlations with chromosomal instability markers including aneuploidy and homologous recombination deficiency. Our comprehensive functional studies demonstrated that this axis critically regulates multiple malignant phenotypes, as evidenced by the rescue of proliferative and metastatic capacities upon HMMR restoration in PSMD14-deficient cells. The synergistic relationship between PSMD14 and HMMR is further supported by our clinical data showing that only concurrent high expression of both molecules significantly worsens patient prognosis.

From a therapeutic perspective, our findings establish PSMD14 as a promising molecular target in LUAD. The potent anti-tumor efficacy of Capzimin, demonstrated both *in vitro* and *in vivo*, validates the therapeutic value of PSMD14 inhibition. This is consistent with previous studies highlighting PSMD14 inhibitors in other malignancies: they have been shown to enhance bortezomib sensitivity and exert synergistic anti-myeloma effects (40), while thiolutin has been identified as a potential treatment in esophageal squamous cell carcinoma by promoting Snail degradation (41). Additionally, PSMD14 inhibitors exhibit antitumor activity and overcome chemoresistance in head and neck squamous cell carcinoma (42). Notably, our study provides mechanistic insights into Capzimin’s action, showing its ability to induce mitochondrial-mediated apoptosis and suppress metastatic potential. Furthermore, the enhanced efficacy observed with combined inhibition of PSMD14 and TGF-β signaling reveals a promising combinatorial strategy that merits further clinical investigation.

The pathogenesis of LUAD involves complex dysregulation of key signaling cascades, with the TGF-β/Smad and PI3K/AKT/mTOR pathways representing two central regulatory axes that collectively govern fundamental cellular processes. The TGF-β/Smad pathway plays a dual role in tumorigenesis, acting as a tumor suppressor in normal and early-stage tissues while promoting invasion, metastasis, and immune evasion in advanced LUAD (43–45). Concurrently, the PI3K/AKT/mTOR axis serves as a master regulator of cell survival, proliferation, metabolism, and protein synthesis, with its hyperactivation frequently observed in LUAD due to mutations in upstream regulators such as EGFR and KRAS (46–48). In the present study, we identified PSMD14 as a common upstream modulator capable of coordinately activating both pathways. Mechanistically, PSMD14 enhanced TGF-β/Smad signaling and concurrently potentiated PI3K/AKT/mTOR activity, thereby synergistically driving malignant phenotypes including proliferation, migration, invasion, and epithelial-mesenchymal transition in LUAD cells. The ability of PSMD14 to simultaneously regulate these two critical pathways provides a mechanistic explanation for its broad impact on LUAD progression, and underscores the therapeutic potential of targeting PSMD14 in tumors exhibiting co-activation of these signaling axes.

Three intriguing observations from this study merit further discussion. First, while our bioinformatic analysis did not show a strong positive correlation between PSMD14 expression and TGF-β signaling in LUAD, both established literature (21) and our experimental data confirm that PSMD14 activates the TGF-β/Smad pathway. This apparent discrepancy may be because PSMD14 regulates the pathway primarily at the post-translational level (e.g., through Smad3 deubiquitination and stabilization) without necessarily altering the core transcriptional output of the pathway captured in mRNA-based correlation analyses. Second, despite ambiguous bioinformatic predictions regarding EMT, our functional experiments—including Western blotting for EMT markers, wound healing, and Transwell assays—uniformly demonstrated that PSMD14 robustly promotes EMT, migration, and invasion in LUAD. The complexity of the EMT regulatory network and the influence of tumor heterogeneity in bulk sequencing data may account for the initial computational ambiguity. Finally, our biochemical assays unequivocally demonstrate that PSMD14 stabilizes the HMMR protein by removing its K63-linked ubiquitin chains. Although we observed concomitant changes in HMMR mRNA levels upon PSMD14 knockdown or overexpression, the core regulatory mechanism underpinning HMMR overexpression in LUAD is its deubiquitination and stabilization by PSMD14. The alterations in mRNA could potentially originate from indirect regulation or downstream feedback effects, the specifics of which warrant future investigation. This finding underscores how oncogenic pathways can employ precise post-translational control to ensure the robust output of key drivers like HMMR during tumorigenesis, a paradigm consistent with reports on other deubiquitinases (49).

While our findings advance the understanding of LUAD pathogenesis, several limitations and future directions should be considered. First, our *in vivo* therapeutic assessment was limited to testing the combination of Capzimin with a TGF-β inhibitor; evaluating its synergy with PI3K/mTOR pathway inhibitors would provide a more comprehensive therapeutic perspective. Furthermore, the xenograft studies were conducted exclusively in male mice to control for hormonal variability, which, while reducing confounding factors, precludes an assessment of potential sex-specific differences in the efficacy of targeting the PSMD14-HMMR axis. At the molecular level, the precise structural basis of the PSMD14-HMMR interaction warrants further investigation. Additionally, although PSMD14-mediated Smad3 deubiquitination is known to promote LUAD metastasis (21), the precise hierarchy and contribution of this pathway relative to other oncogenic signaling axes, such as PI3K/AKT/mTOR, in driving specific malignant phenotypes remain to be clarified. Future studies should also explore potential crosstalk between PSMD14 and immune checkpoint molecules, given its association with an immunosuppressive microenvironment.

In conclusion, our work establishes PSMD14 as a vital regulator of LUAD progression through its dual functions as a deubiquitinating enzyme for HMMR and a coordinator of oncogenic signaling pathways. These findings not only provide insights into LUAD biology but also offer translational opportunities for biomarker development and targeted therapy. The PSMD14-HMMR axis represents a promising therapeutic target, particularly in patients exhibiting concurrent overexpression of both molecules who may benefit most from pathway-specific interventions.

## 5 Conclusions

This study elucidates the molecular mechanism by which PSMD14 drives LUAD progression through K63-linked deubiquitination and stabilization of HMMR. As a key oncoprotein, PSMD14 promotes malignant phenotypes including proliferation, migration, and invasion by concurrently activating both TGF-β/Smad and PI3K/AKT/mTOR signaling pathways. Importantly, the PSMD14 inhibitor Capzimin demonstrates significant anti-tumor activity, validating the therapeutic potential of targeting the PSMD14-HMMR axis. These findings not only establish the PSMD14-HMMR axis as a promising diagnostic biomarker and therapeutic target in LUAD but also provide an experimental foundation for developing precision therapeutics against this pathway.

## Data Availability

The original contributions presented in the study are included in the article/**Supplementary Material**. Further inquiries can be directed to the corresponding author.

## Supplementary material

Supplementary Figure 1 Screening and validation of HMMR as a key downstream target of PSMD14 in LUAD. **(A)** mRNA expression levels of selected PSMD14-interacting proteins (NCL, SERBP1, SMC2, IGF2BP1, PKM, HMMR) in LUAD tumors and adjacent normal tissues from the GEPIA database. **(B)** Kaplan-Meier survival analysis of LUAD patients from the GEPIA database, stratified by high or low expression of the indicated genes. **(C)** Correlation analysis between PSMD14 and candidate gene expression in the TCGA-LUAD cohort. Pearson correlation coefficients are indicated. **(D)** Western blot analysis of the indicated proteins in LUAD cells after PSMD14 knockdown. **(E)** Scatter plot and statistical analysis of the correlation between PSMD14 and HMMR protein levels across different LUAD cell lines (Pearson r = 0.804, p = 0.029). **(F)** H1975 cells overexpressing PSMD14 were treated with MG132, and the ubiquitination of HMMR was assessed by immunoprecipitation using antibodies against K63-linked and K48-linked ubiquitin chains.

Supplementary Figure 2 PSMD14 knockdown inhibits proliferation, migration, and invasion in PC9 cells. **(A)** Cell viability of PC9 cells measured by CCK-8 assay after PSMD14 knockdown. **(B)** Colony formation ability of PC9 cells following PSMD14 knockdown. **(C)** Proliferation of PC9 cells assessed by EdU assay upon PSMD14 knockdown. **(D)** Migration capability of PC9 cells evaluated by wound healing assay after PSMD14 knockdown. **(E)** Migration and invasion of PC9 cells determined by Transwell assay following PSMD14 knockdown. **(F)** Correlation analysis between PSMD14 expression and genomic instability scores. **P* < 0.05, ** *P* < 0.01, ****P* < 0.001.
