# Supplementary material for: PSMD14 drives lung adenocarcinoma progression through HMMR stabilization and dual activation of TGF-β/Smad and PI3K/AKT/mTOR signaling
Source: Front Immunol. 2025 Dec 19;16:1720799. doi: 10.3389/fimmu.2025.1720799 (PMC12757429; doi:10.3389/fimmu.2025.1720799)
Supplement: Supplementary Figure 1 — Screening and validation of HMMR as a key downstream target of PSMD14 in LUAD. (A) mRNA expression levels of selected PSMD14-interacting proteins (NCL, SERBP1, SMC2, IGF2BP1, PKM, HMMR) in LUAD tumors and adjacent normal tissues from the GEPIA database. (B) Kaplan-Meier survival analysis of LUAD patients from the GEPIA database, stratified by high or low expression of the indicated genes. (C) Correlation analysis between PSMD14 and candidate gene expression in the TCGA-LUAD cohort. Pearson correlation coefficients are indicated. (D) Western blot analysis of the indicated proteins in LUAD cells after PSMD14 knockdown. (E) Scatter plot and statistical analysis of the correlation between PSMD14 and HMMR protein levels across different LUAD cell lines (Pearson r = 0.804, p = 0.029). (F) H1975 cells overexpressing PSMD14 were treated with MG132, and the ubiquitination of HMMR was assessed by immunoprecipitation using antibodies against K63-linked and K48-linked ubiquitin chains. [file DataSheet1.zip › Data sheet 1/Supplementary Material.pdf]

Figure 4B

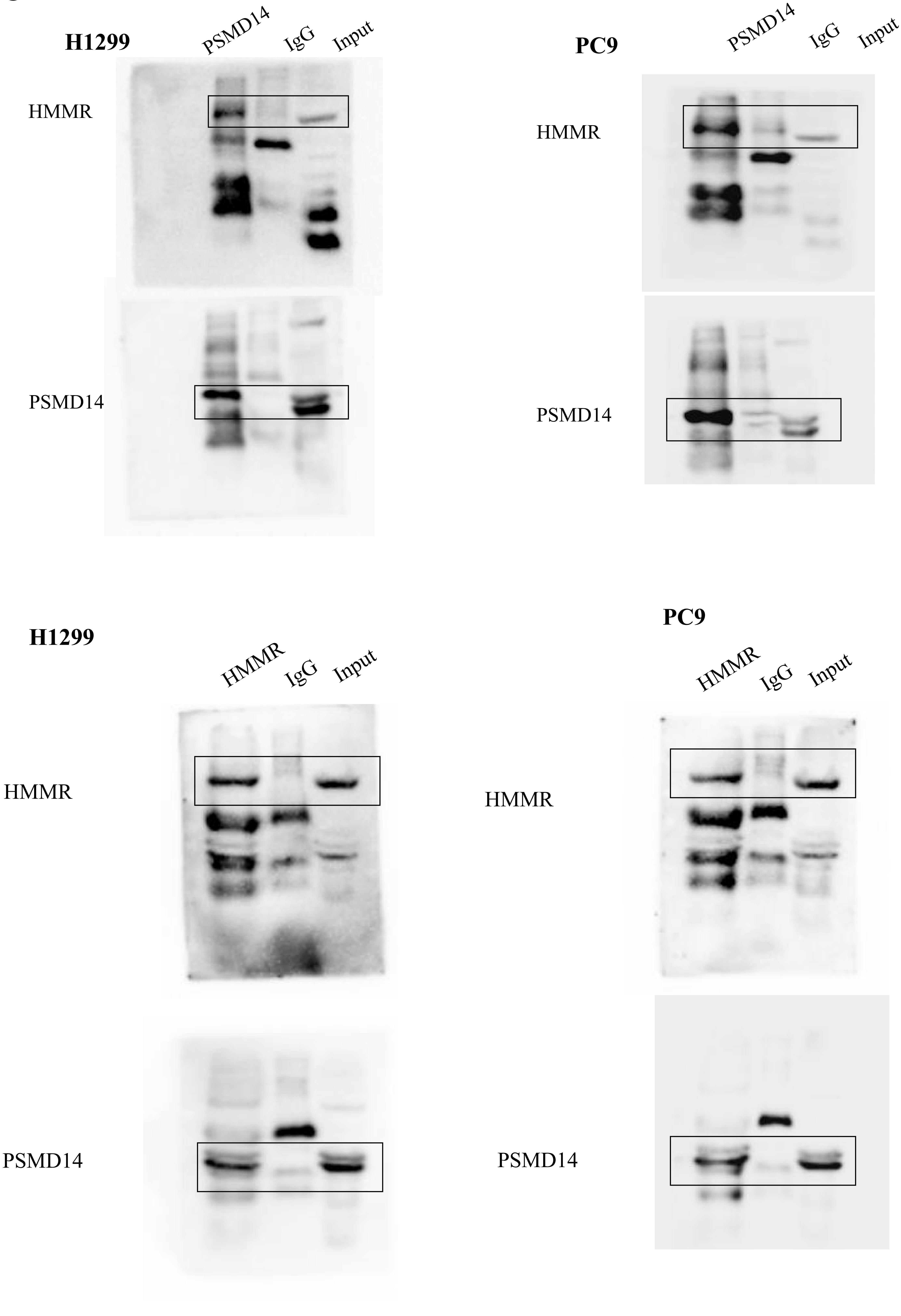
H1299
PSMD14
IgG
Input
HMMR
PSMD14
PC9
PSMD14
IgG
Input
HMMR
PSMD14
H1299
HMMR
IgG
Input
HMMR
PSMD14
PC9
HMMR
IgG
Input
HMMR
PSMD14

Figure 4C

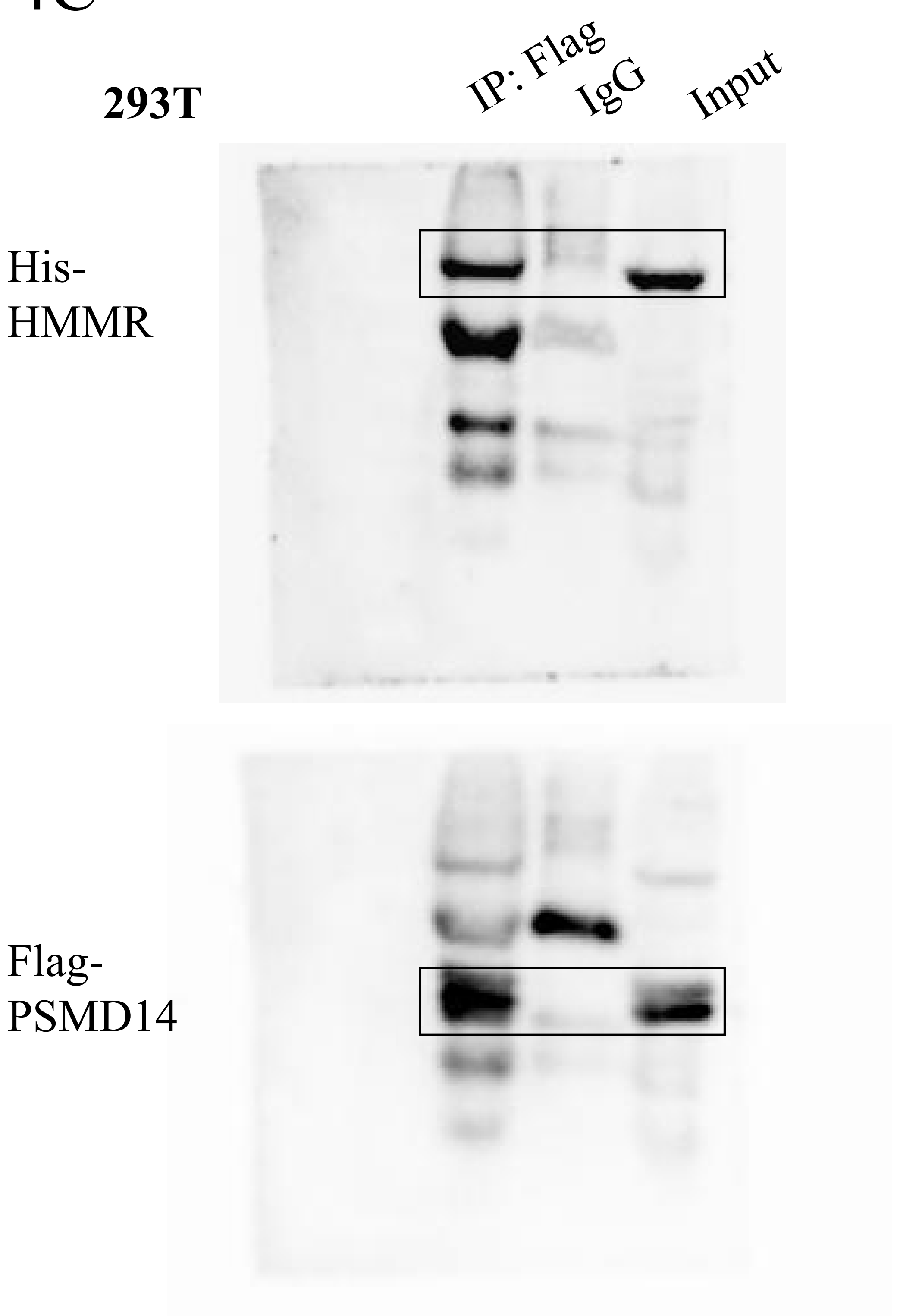

Figure 4D

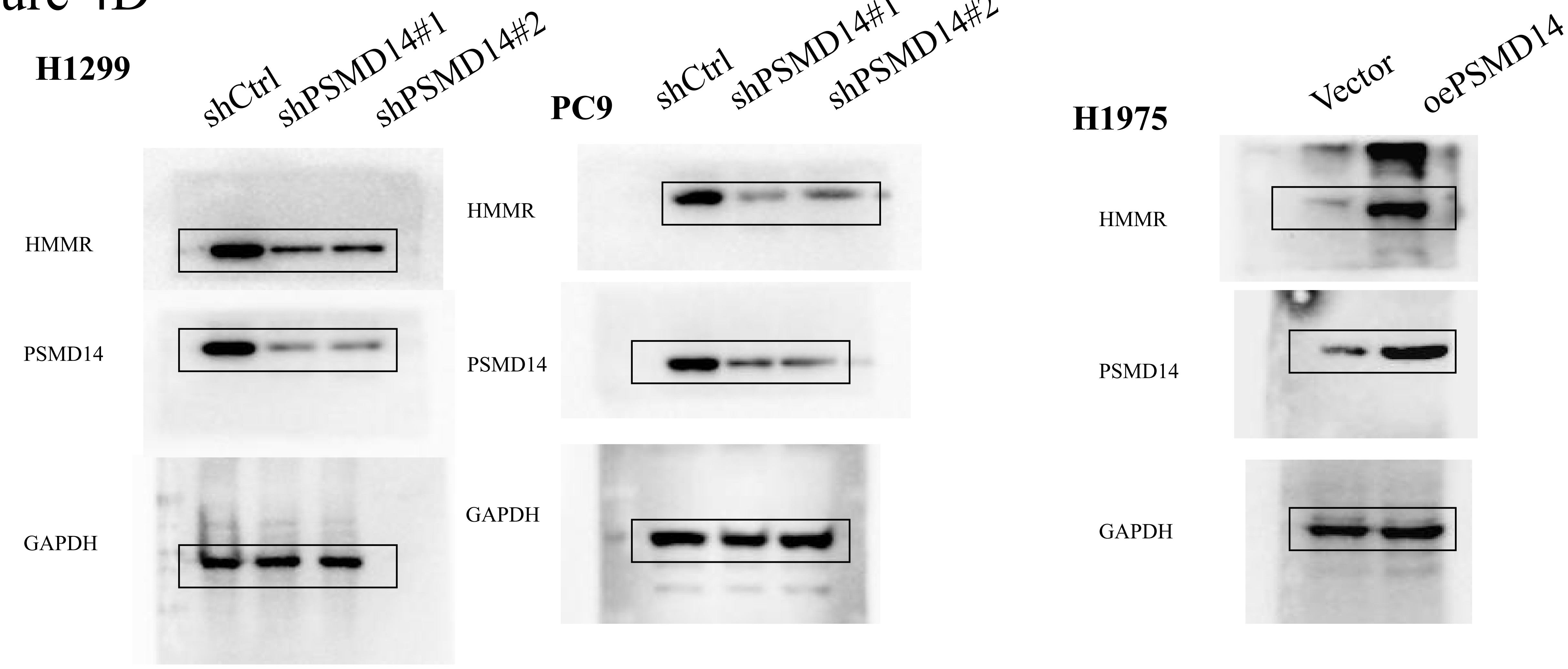

Figure 5A

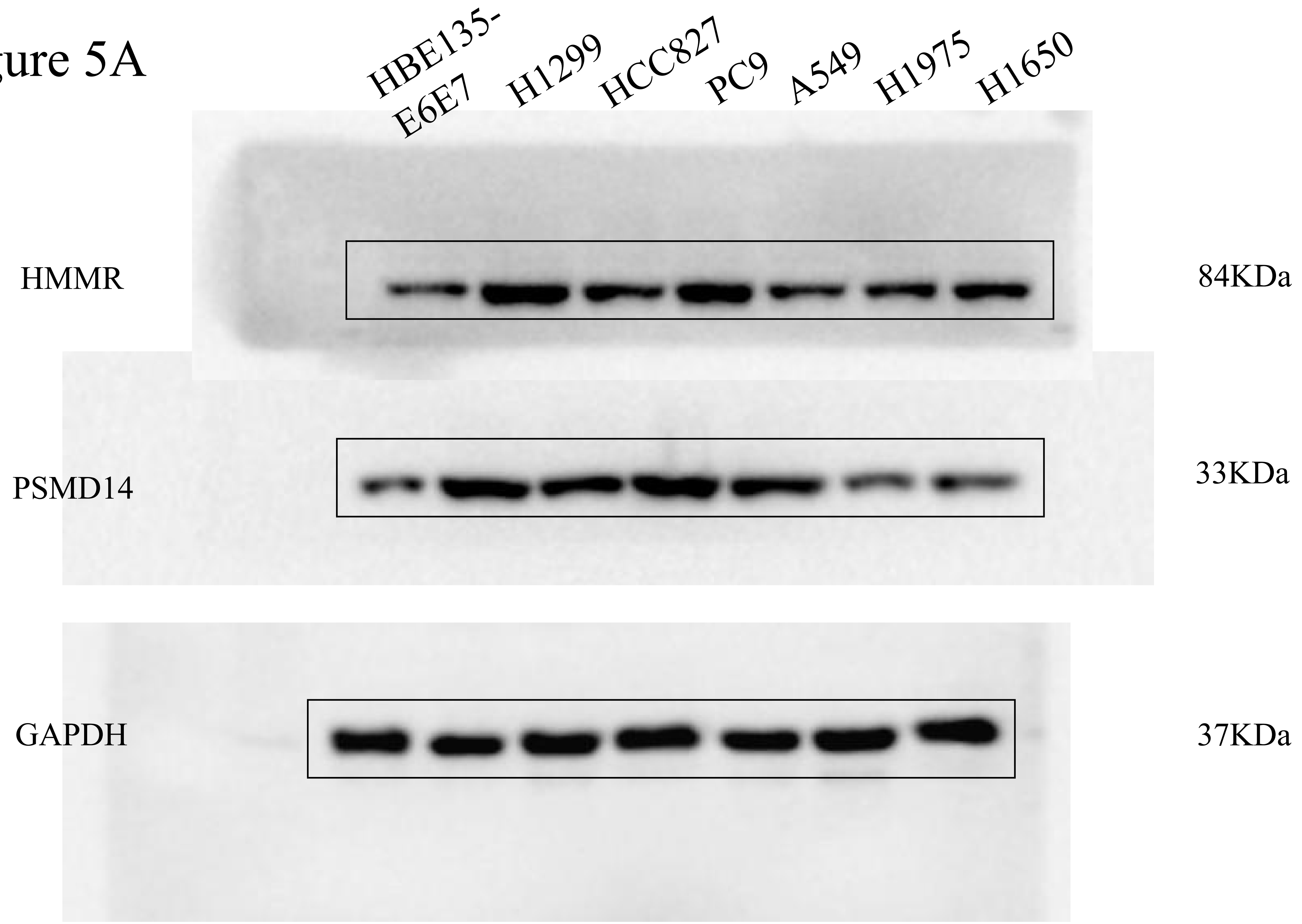

Figure 5B

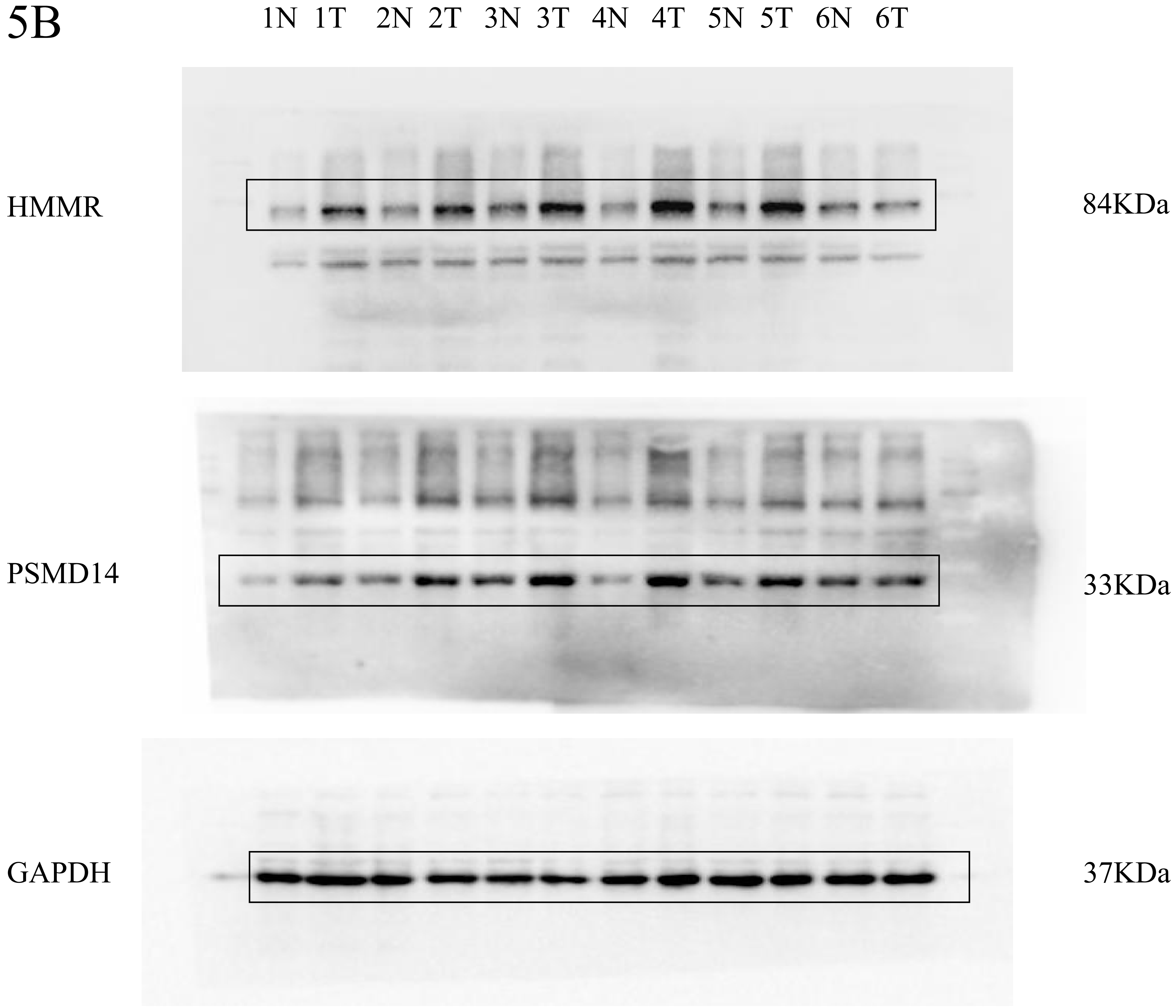

Figure 5B

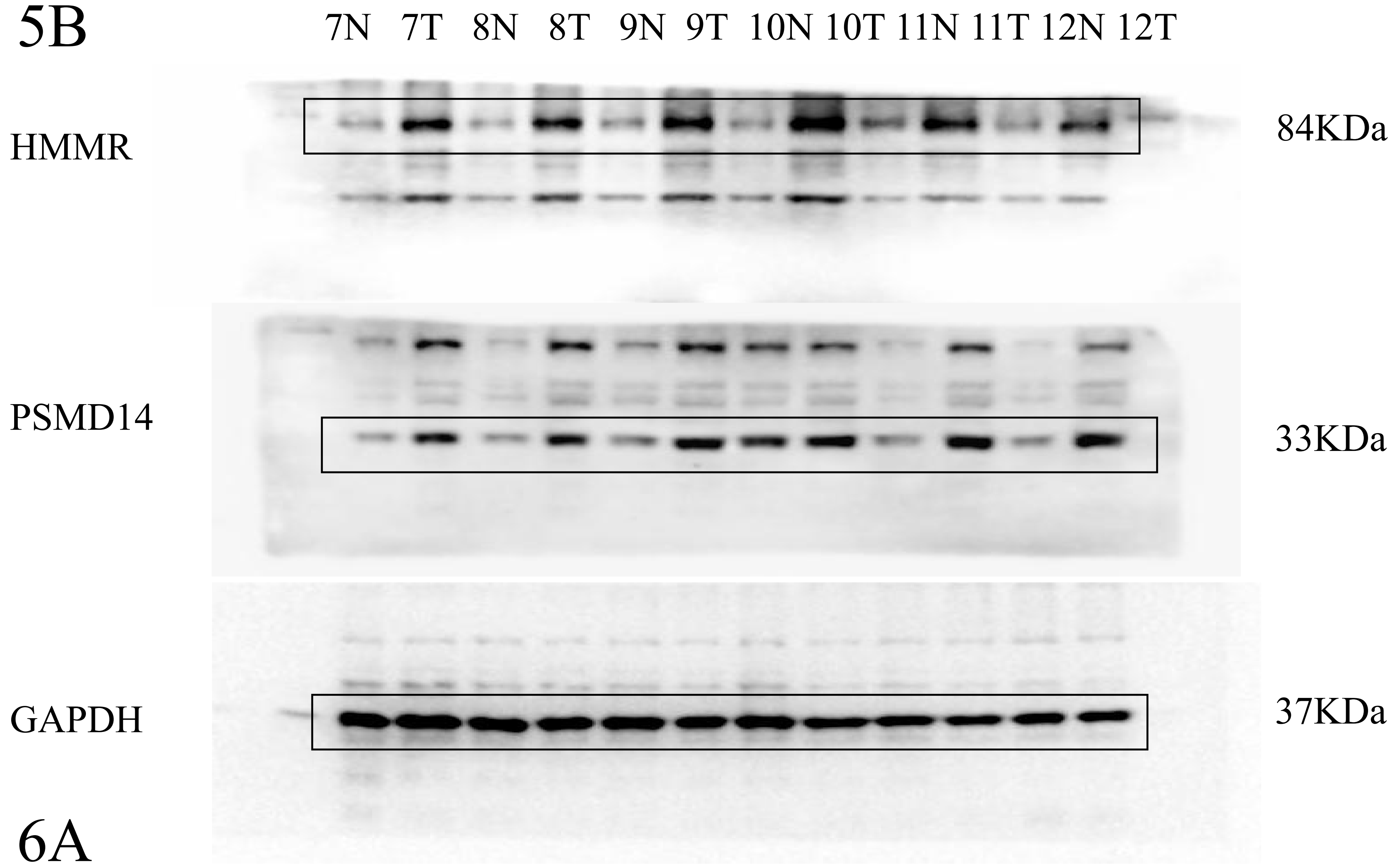

Figure 6A

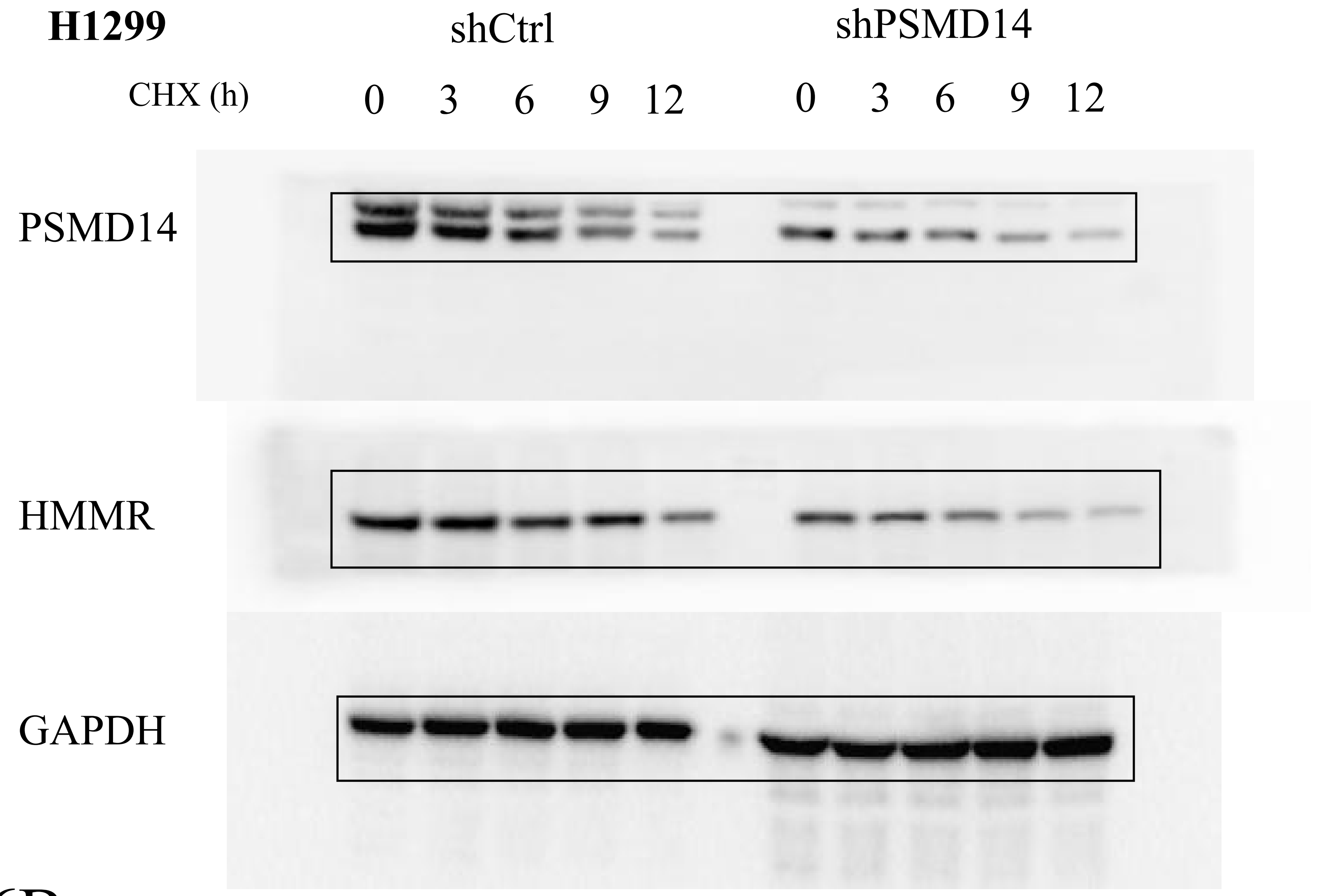

Figure 6B

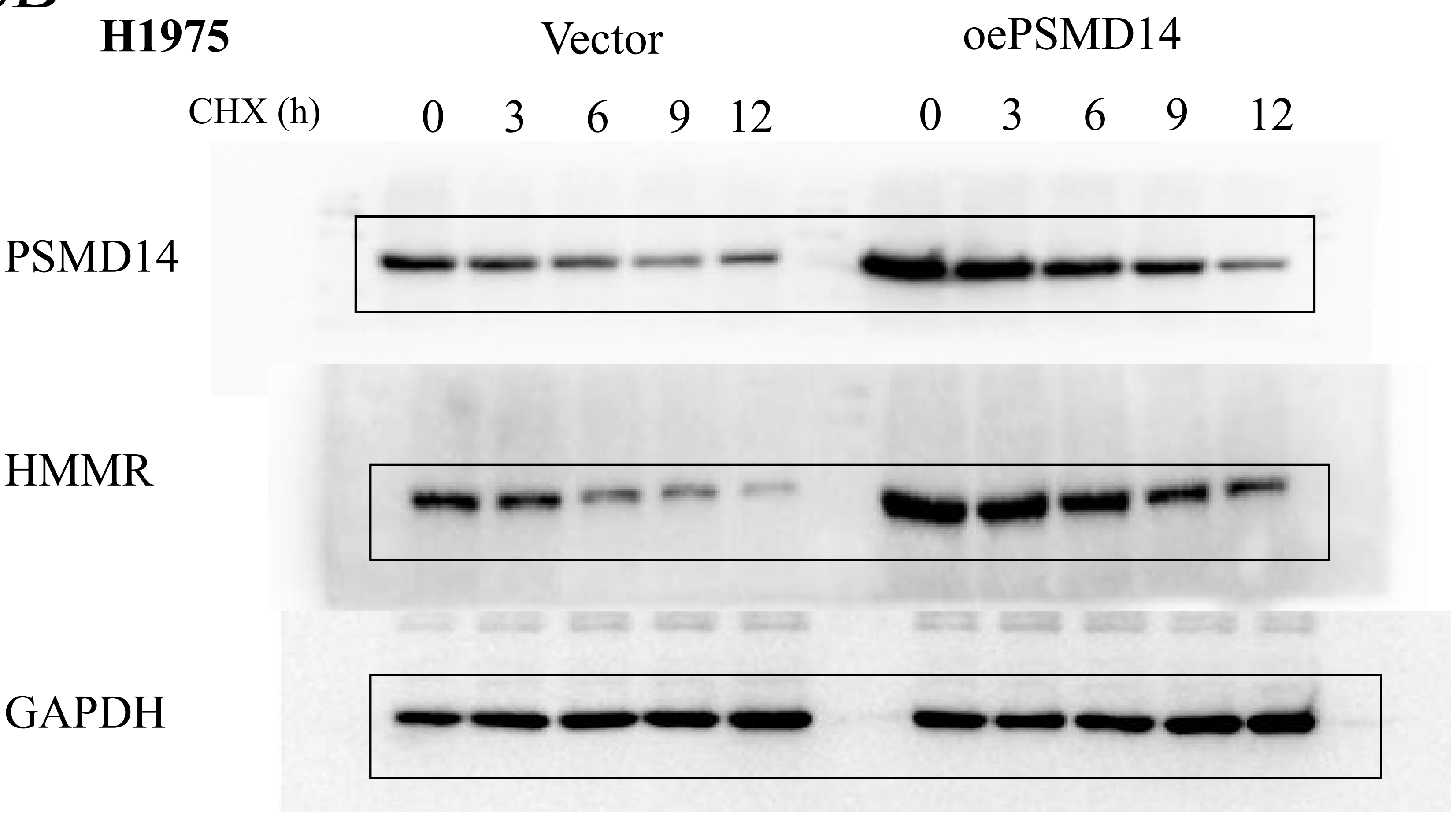

Figure 6C

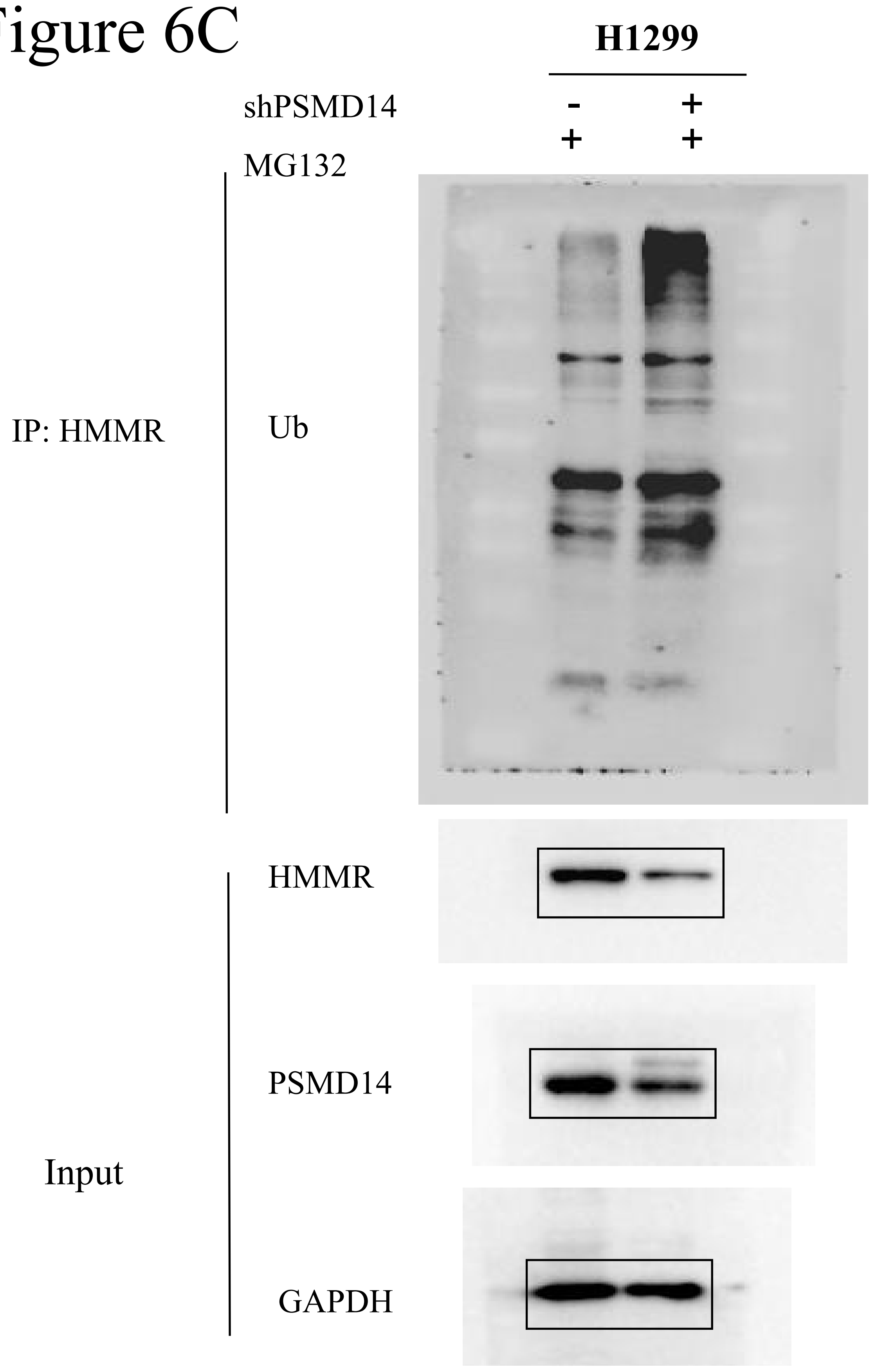

Figure 6D

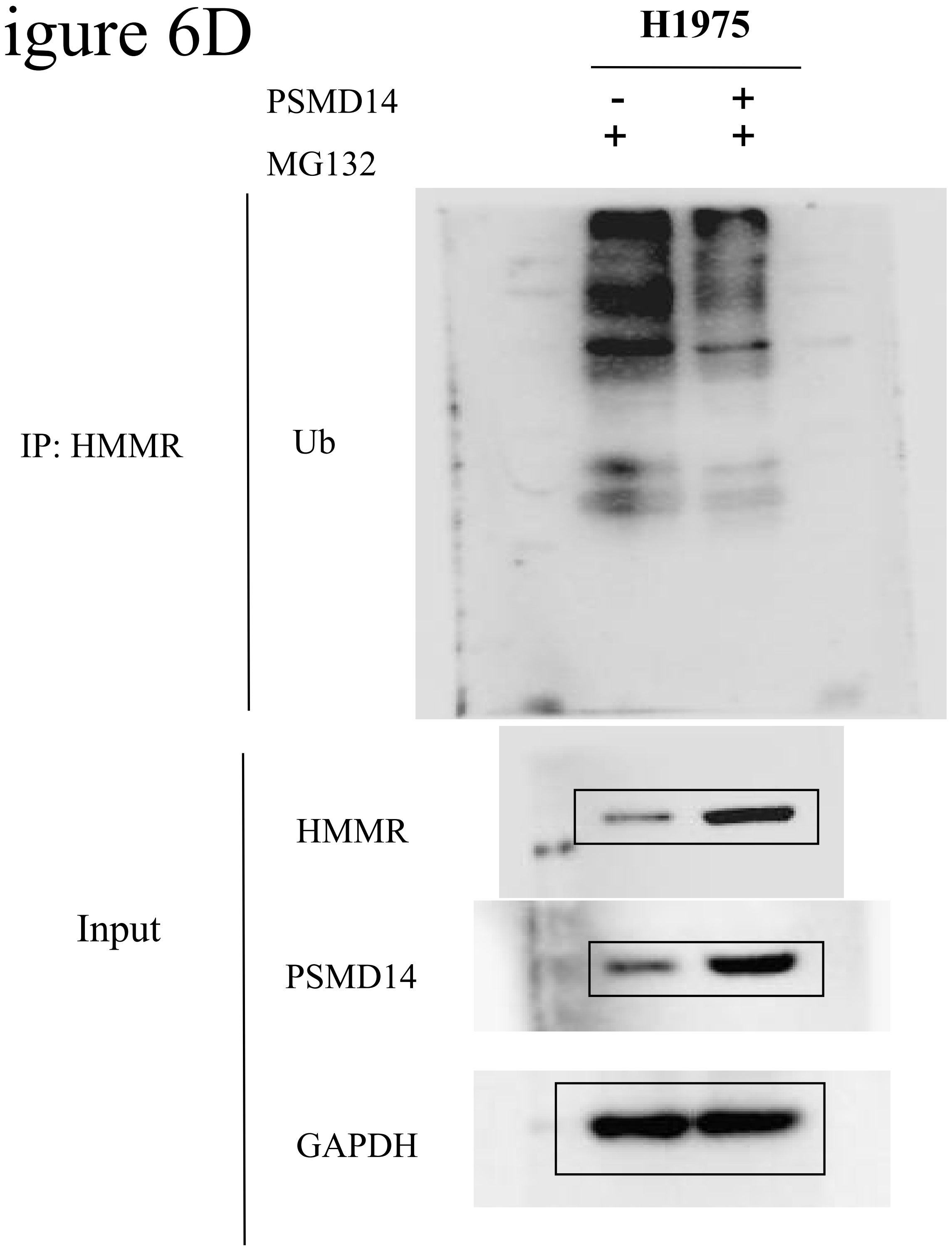

Figure 6E

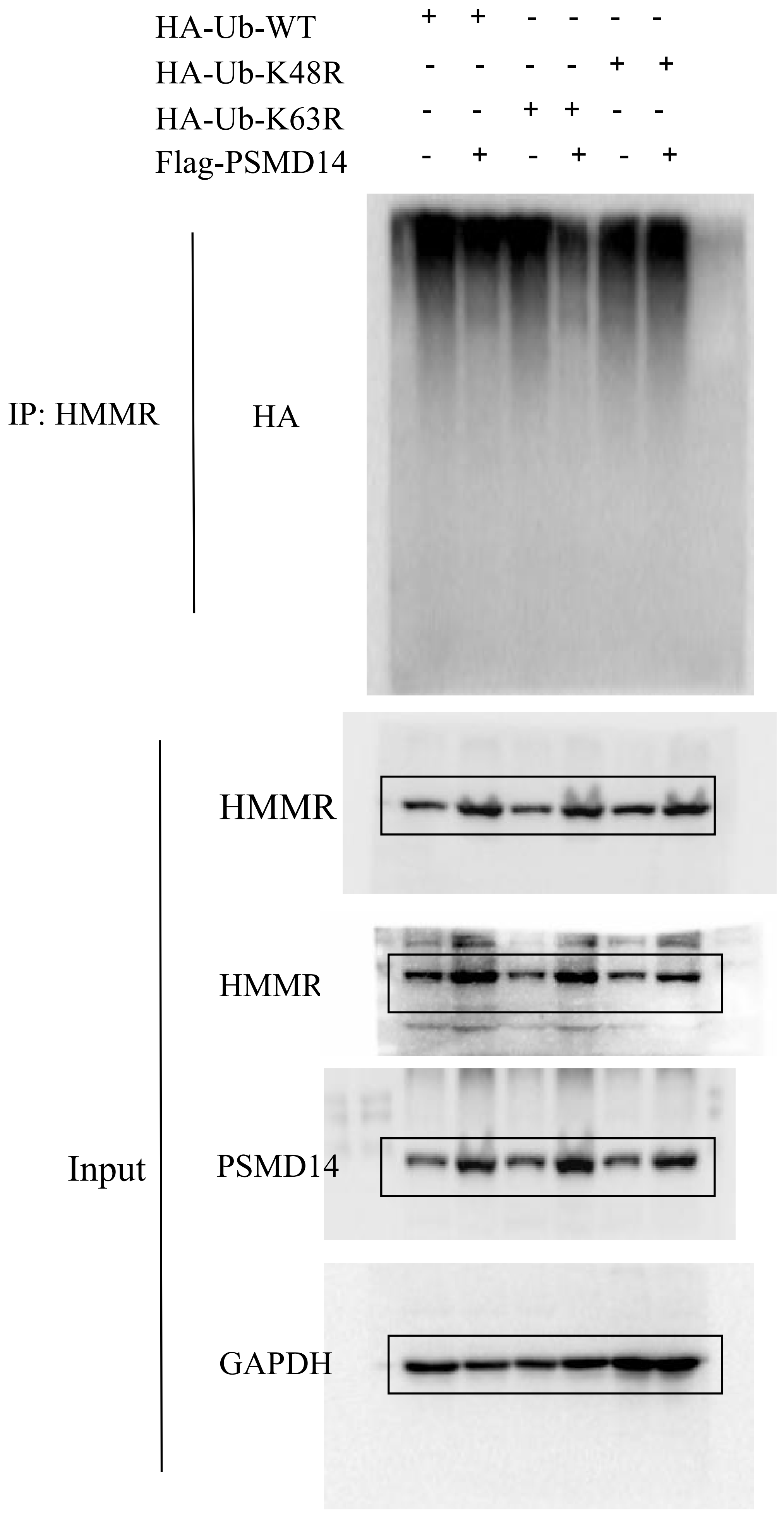

Figure 7G

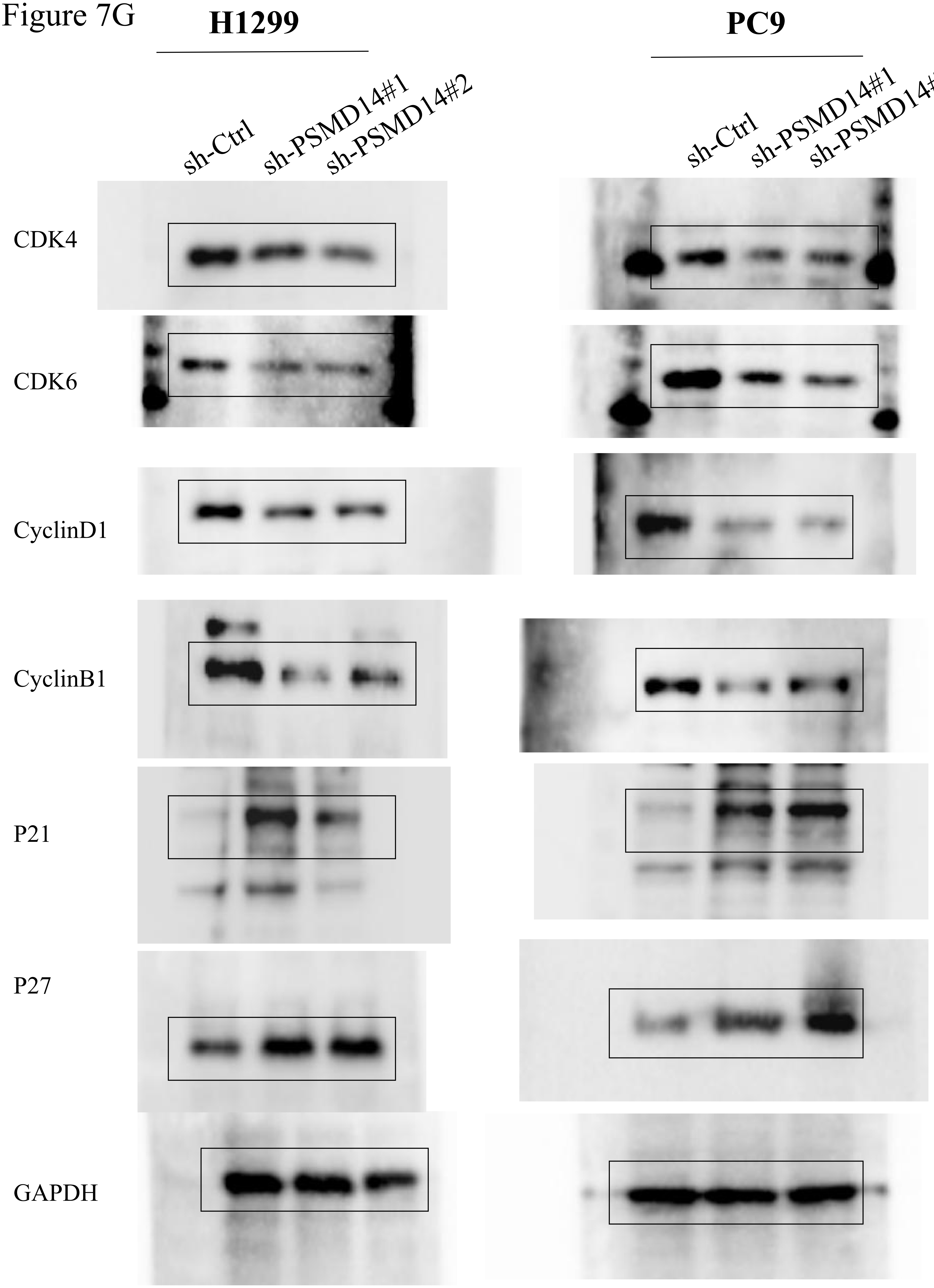
H1299
PC9
sh-Ctrl
sh-PSMD14#1
sh-PSMD14#2
sh-Ctrl
sh-PSMD14#1
sh-PSMD14#2
CDK4
CDK6
CyclinD1
CyclinB1
P21
P27
GAPDH

Figure 8C

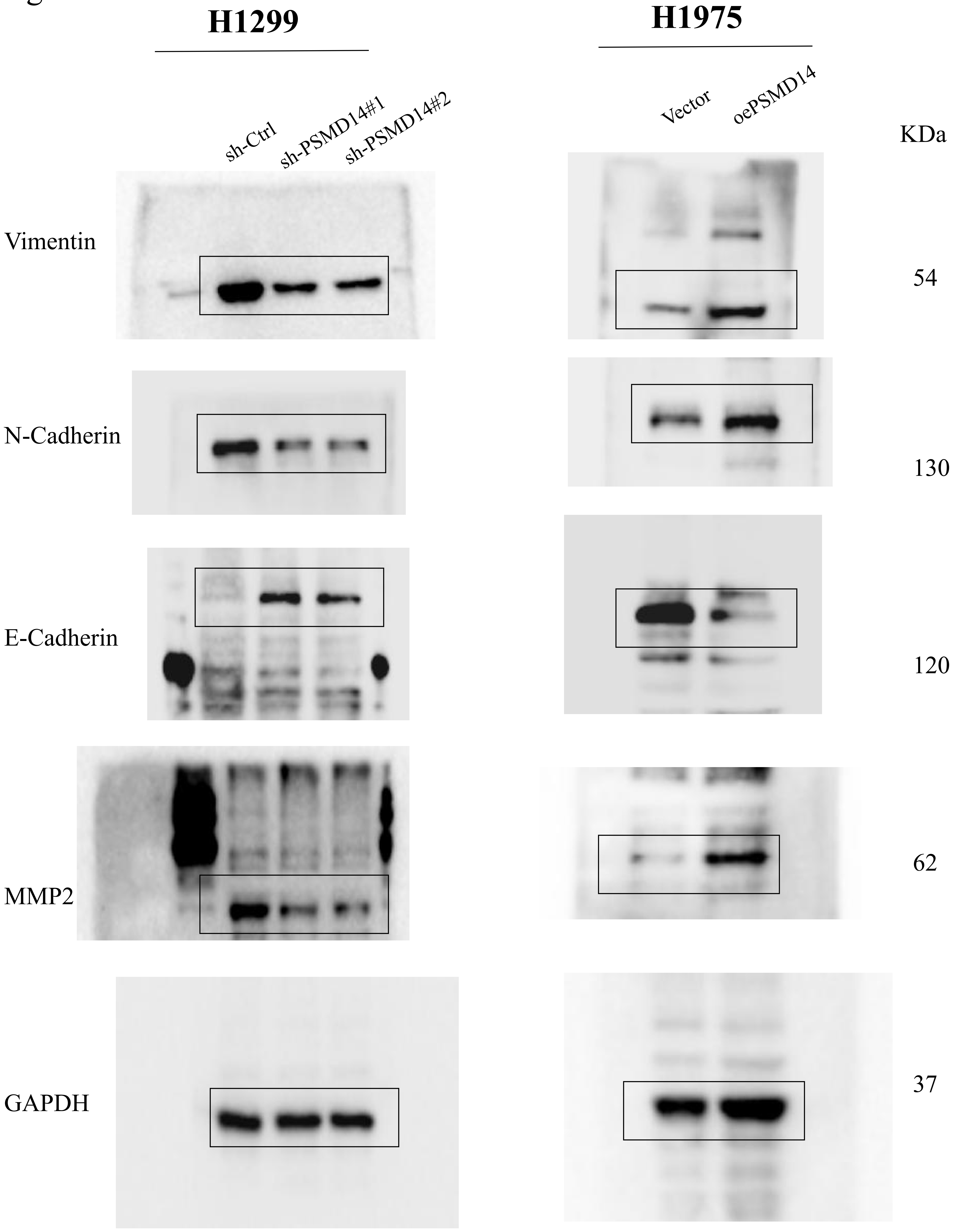
H1299
H1975
sh-Ctrl
sh-PSMD14#1
sh-PSMD14#2
Vector
oePSMD14
KDa
Vimentin
54
N-Cadherin
130
E-Cadherin
120
MMP2
62
GAPDH
37

Figure 8D

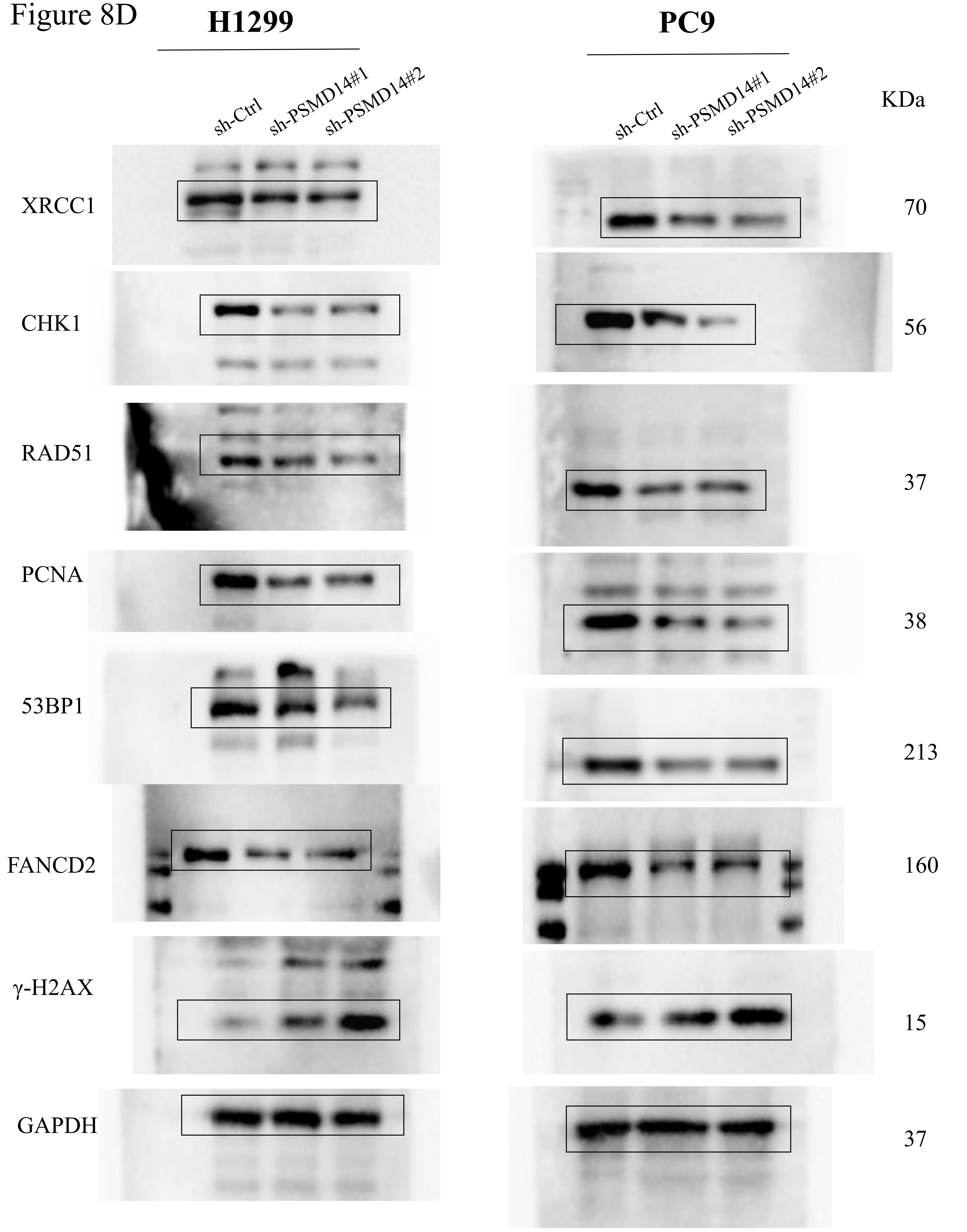
H1299
PC9
sh-Ctrl
sh-PSMD14#1
sh-PSMD14#2
KDa
XRCC1
CHK1
RAD51
PCNA
53BP1
FANCD2
γ-H2AX
GAPDH
70
56
37
38
213
160
15
37

Figure 11E

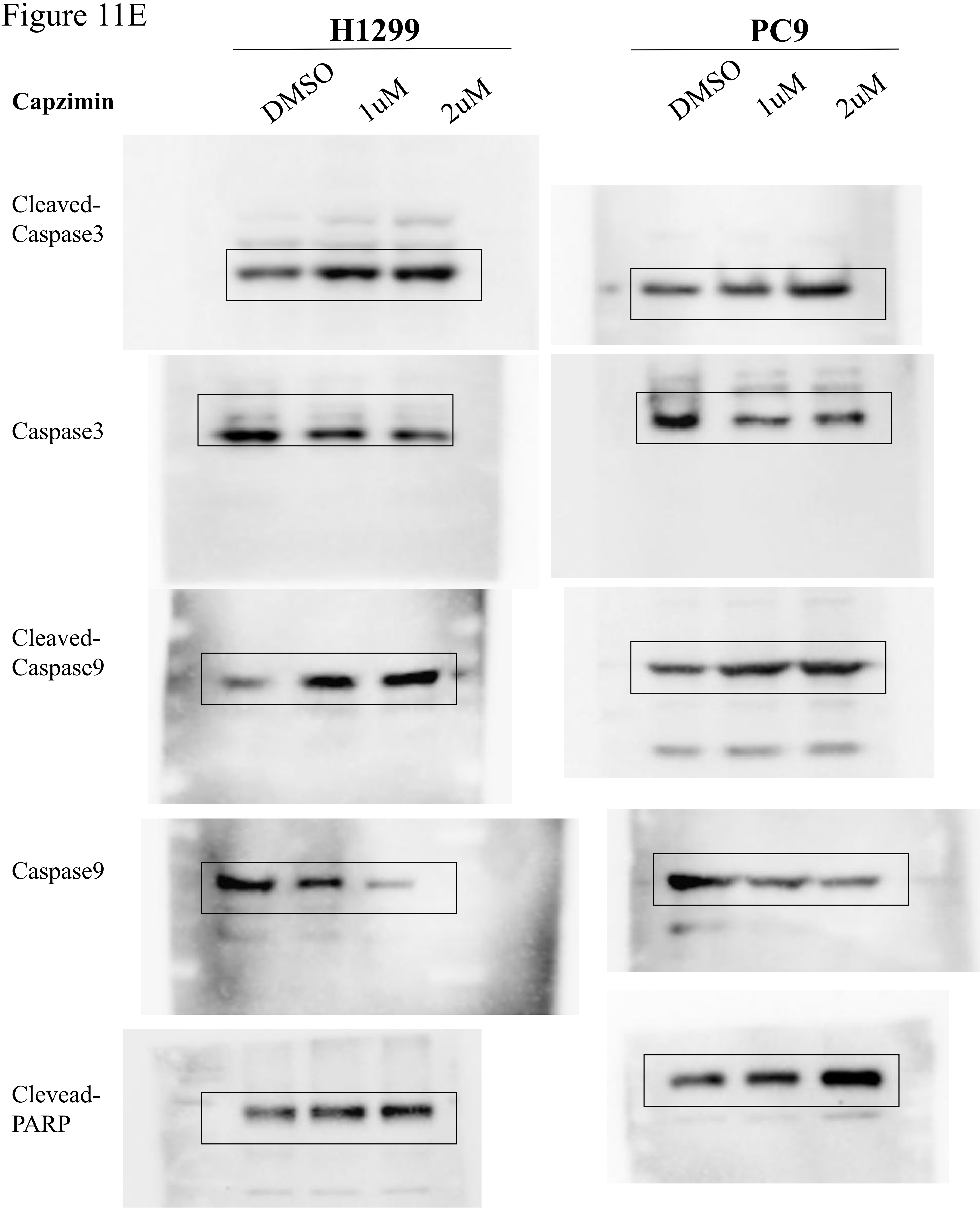
H1299
PC9
Capzimin
DMSO
1uM
2uM
DMSO
1uM
2uM
Cleaved-
Caspase3
Caspase3
Cleaved-
Caspase9
Caspase9
Clevead-
PARP

Figure 11E

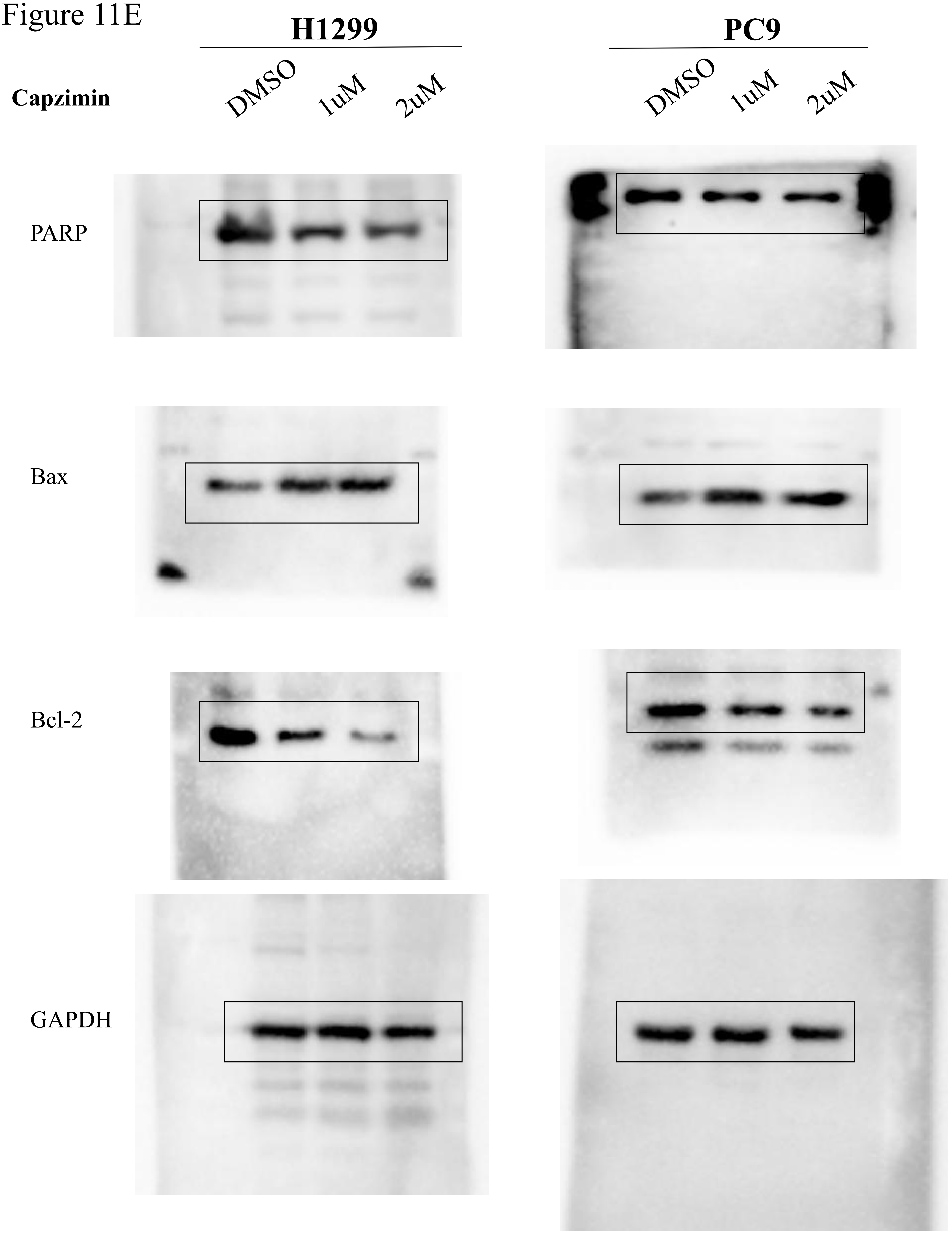
H1299
PC9
Capzimin
DMSO
1uM
2uM
DMSO
1uM
2uM
PARP
Bax
Bcl-2
GAPDH

Figure 12B

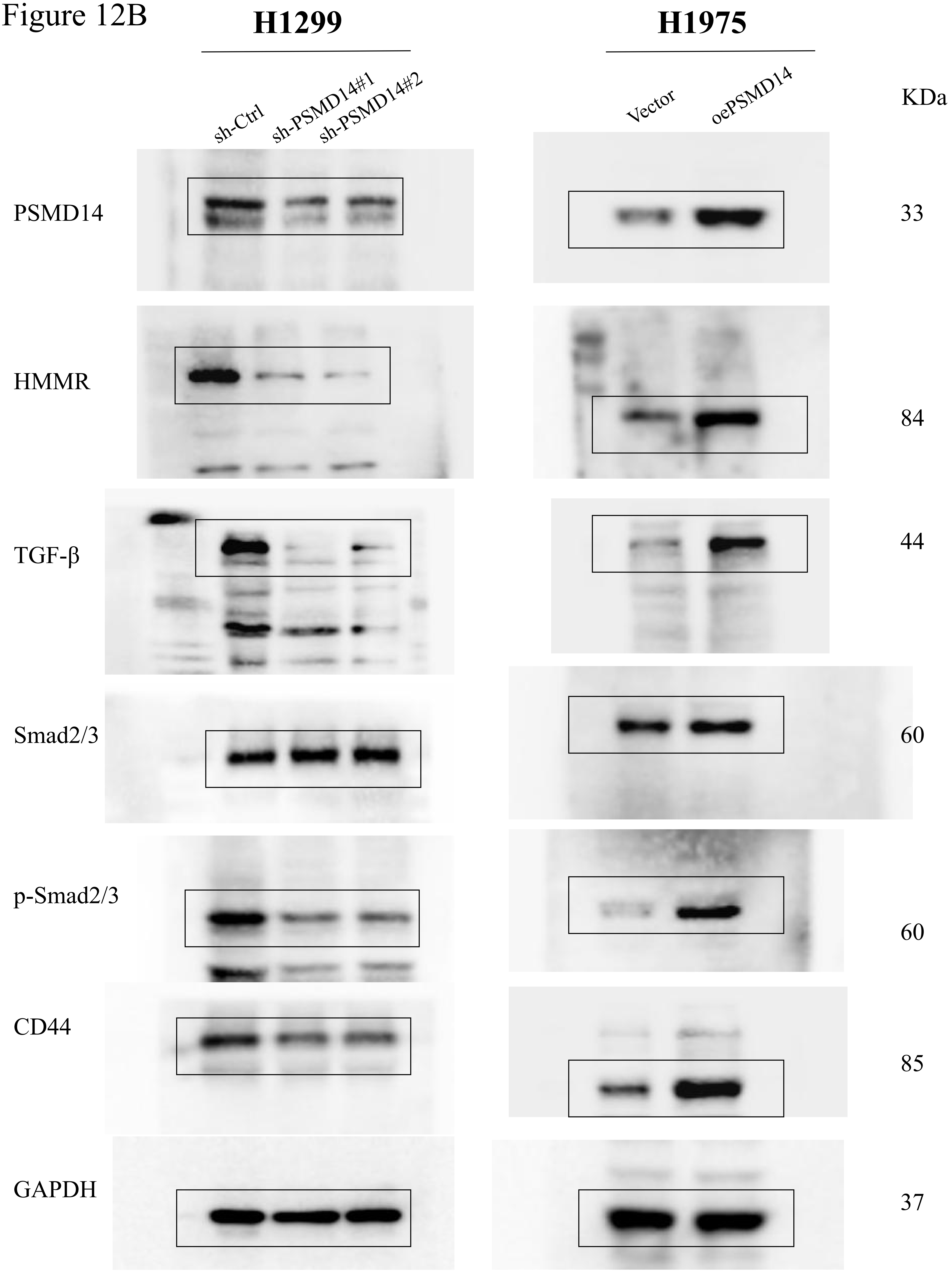
H1299
H1975
sh-Ctrl
sh-PSMD14#1
sh-PSMD14#2
Vector
oePSMD14
KDa
PSMD14
33
HMMR
84
TGF-β
44
Smad2/3
60
p-Smad2/3
60
CD44
85
GAPDH
37

Figure 12C

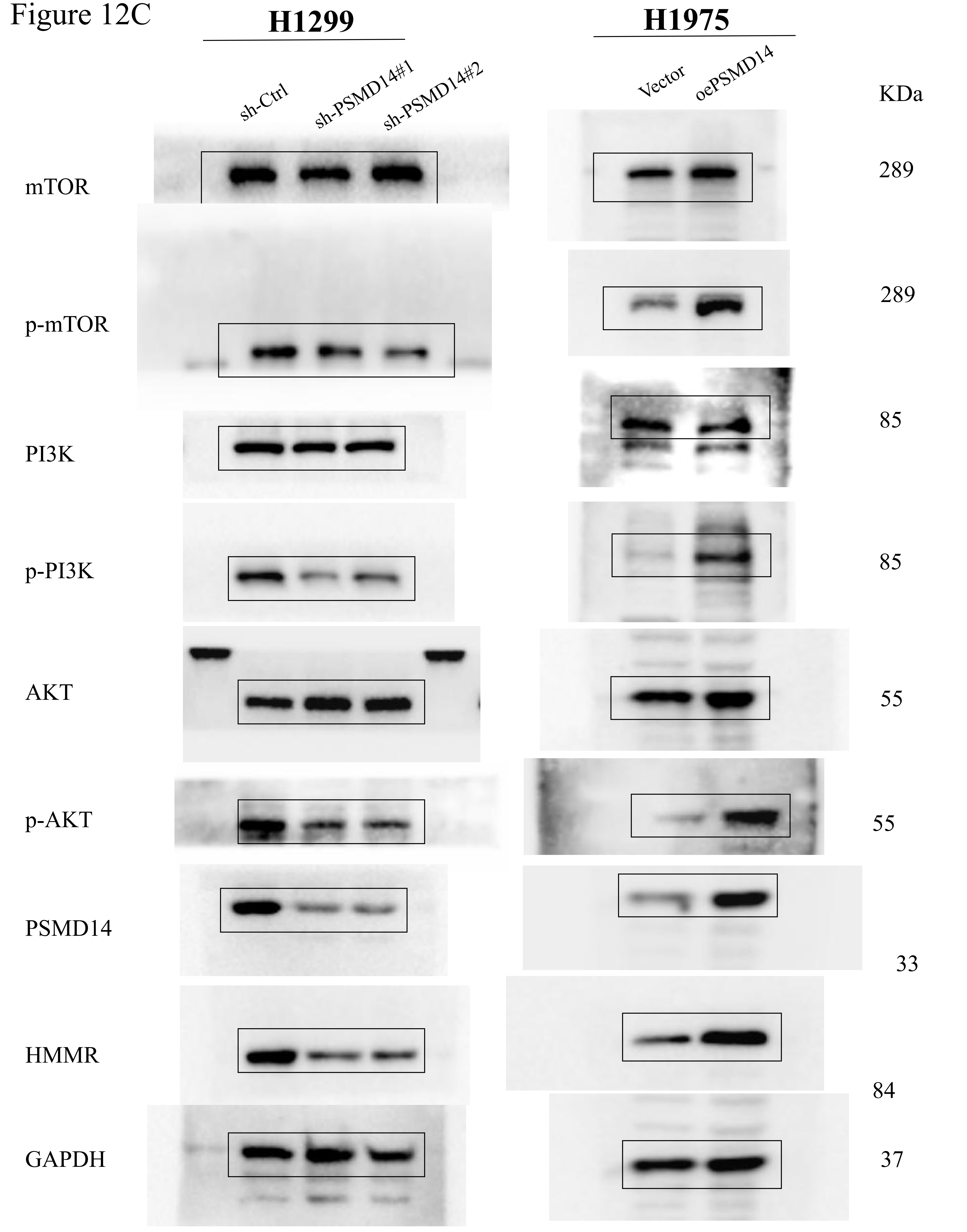

H1299
H1975
sh-Ctrl
sh-PSMD14#1
sh-PSMD14#2
Vector
oePSMD14
KDa
mTOR
p-mTOR
PI3K
p-PI3K
AKT
p-AKT
PSMD14
HMMR
GAPDH
289
289
85
85
55
55
33
84
37

Figure S1

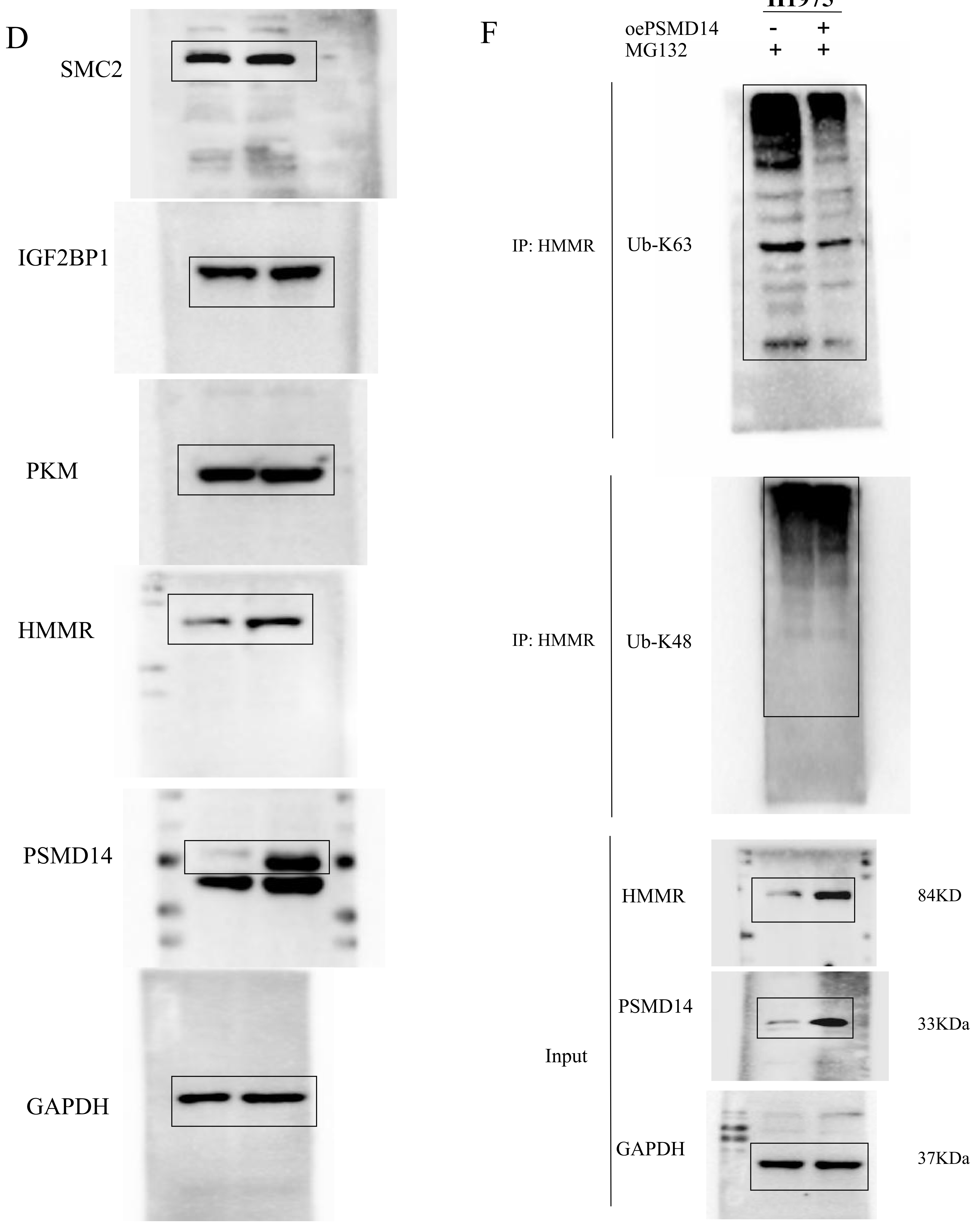
D
SMC2
IGF2BP1
PKM
HMMR
PSMD14
GAPDH
F
H1975
oePSMD14 - +
MG132 + +
IP: HMMR
Ub-K63
IP: HMMR
Ub-K48
Input
HMMR
84KD
PSMD14
33KDa
GAPDH
37KDa
