# Supplementary material for: PSMD14 drives lung adenocarcinoma progression through HMMR stabilization and dual activation of TGF-β/Smad and PI3K/AKT/mTOR signaling
Source: Front Immunol. 2025 Dec 19;16:1720799. doi: 10.3389/fimmu.2025.1720799 (PMC12757429; doi:10.3389/fimmu.2025.1720799)
Supplement: Supplementary Figure 1 — Screening and validation of HMMR as a key downstream target of PSMD14 in LUAD. (A) mRNA expression levels of selected PSMD14-interacting proteins (NCL, SERBP1, SMC2, IGF2BP1, PKM, HMMR) in LUAD tumors and adjacent normal tissues from the GEPIA database. (B) Kaplan-Meier survival analysis of LUAD patients from the GEPIA database, stratified by high or low expression of the indicated genes. (C) Correlation analysis between PSMD14 and candidate gene expression in the TCGA-LUAD cohort. Pearson correlation coefficients are indicated. (D) Western blot analysis of the indicated proteins in LUAD cells after PSMD14 knockdown. (E) Scatter plot and statistical analysis of the correlation between PSMD14 and HMMR protein levels across different LUAD cell lines (Pearson r = 0.804, p = 0.029). (F) H1975 cells overexpressing PSMD14 were treated with MG132, and the ubiquitination of HMMR was assessed by immunoprecipitation using antibodies against K63-linked and K48-linked ubiquitin chains. [file DataSheet1.zip › Data sheet 1/Supplementary Methods.docx]

**3.16 Bioinformatics analysis**

**3.16.1 Data Integration and Normalization**
To expand the normal tissue sample size, transcriptomic data from GTEx normal samples were integrated and paired with LUAD tumor samples from TCGA, with both datasets quantified in transcripts per million (TPM). Expression values were standardized into dimensionless Z-scores using the formula (x – μ)/σ to minimize batch effects and scale variations. Outliers with Z-scores below -3 or above 3 were excluded from subsequent analyses.

**3.16.2 Diagnostic Performance Evaluation**
The diagnostic performance of PSMD14 in discriminating LUAD tumors from normal tissues was evaluated using receiver operating characteristic (ROC) analysis implemented with the R package “pROC”. The area under the curve (AUC) and its 95% confidence interval (95% CI) were calculated. An AUC closer to 1 indicates superior diagnostic ability, with values above 0.7, 0.8, and 0.9 generally interpreted as indicating moderate, good, and excellent performance, respectively. Calibration curves were plotted to assess the agreement between predicted probabilities and observed outcomes, and the Hosmer–Lemeshow test was used to evaluate deviations from ideal model calibration.

**3.16.3 Protein Expression Analysis**
Protein expression data for LUAD were obtained from the Clinical Proteomic Tumor Analysis Consortium (CPTAC) (https://proteomics.cancer.gov/programs/cptac), which provides comprehensive proteomic profiles for multiple cancer types along with matched normal tissues. The Wilcoxon rank-sum test was applied to compare PSMD14 protein expression levels between tumor and normal groups.

**3.16.4 Stratification and Clinical Association Analysis**

A comparison of PSMD14 expression across tumor stages in the TCGA-LUAD cohort was performed using the Wilcoxon rank-sum test. Patients were categorized into early-stage (I/II) and late-stage (III/IV) groups for this analysis.

Patients from the TCGA-LUAD and GSE31210 cohorts were stratified into four quartiles (Q1–Q4) based on PSMD14 expression levels, with Q1 and Q4 representing the highest and lowest 25% of expressers, respectively. The chi-square test was then employed to assess the significance of clinical characteristic distributions across these expression-based subgroups.

**3.16.5 Survival Analysis**
Survival data for LUAD patients were obtained from public repositories, including TCGA-LUAD, CPTAC, and the GEO database (GSE30219, GSE31210, GSE42127, GSE68465, and GSE8894). These integrated datasets were leveraged for subsequent survival analysis. Univariate Cox regression and Kaplan–Meier survival analyses were performed using the R package “survival”. The optimal expression cutoff for dichotomizing patients into high- and low-expression groups was determined using the “survminer” package, with the constraint that each group comprise no less than 30% of the cohort. Differences in survival between groups were assessed using the log-rank test via the survfit function. Hazard ratios (HRs) and 95% CIs were derived from univariate Cox models to quantify the association between PSMD14 expression and overall survival. An HR > 1 indicates increased risk, whereas an HR < 1 suggests a protective effect.

**3.16.6 Functional States and Pathway Activity Analysis**
To investigate functional states of cancer cells, data on 14 distinct states were retrieved from the CancerSEA database. Combined z-scores for these gene sets were computed using the z-score parameter algorithm in the R package GSVA, as proposed by Lee et al[1-3], which infers pathway activity based on the expression of signature genes. The resulting scores were further standardized using the scale function to generate normalized gene set scores. Pearson correlation analysis was then performed to assess the relationship between individual gene expression and each functional state score.

Protein expression data derived from reverse-phase protein arrays (RPPA) were obtained from The Cancer Proteome Atlas (TCPA). Pathway activity scores for ten cancer-related signaling pathways[4] (TSC/mTOR, RTK, RAS/MAPK, PI3K/AKT, Hormone ER, Hormone AR, EMT, DNA Damage Response, Cell Cycle, and Apoptosis) were computed based on established methodologies. Spearman correlation analysis between the target gene expression and these pathway activity scores was performed using the cor.test function in R, yielding both correlation coefficients and associated p-values.

Pathway activity was inferred using the R package “PROGENy” (version 1.10.0)[5, 6], which employs a curated set of pathway-responsive genes derived from perturbation experiments to estimate the activity of 14 signaling pathways: Androgen, EGFR, Estrogen, Hypoxia, JAK-STAT, MAPK, NF-κB, p53, PI3K, TGF-β, TNF-α, Trail, VEGF, and WNT. The resulting pathway scores were Z-score normalized using the formula (x–μ)/σ. Subsequently, samples were dichotomized into high- and low-expression groups based on the median expression of the gene of interest, and differences in pathway activity between these groups were compared to infer the potential mechanistic involvement of the gene.

**3.16.7 Drug Sensitivity Correlation Analysis**

Spearman correlation analysis was employed to assess the association between PSMD14 expression and drug sensitivity, quantified by the area under the dose-response curve (AUC), using data from the CTRP (https://portals.broadinstitute.org/ctrp.v2.1/) and PRISM (https://www.theprismlab.org) databases. An inverse correlation (negative Spearman's ρ) indicated that higher PSMD14 expression was associated with increased drug sensitivity, whereas a positive correlation suggested greater drug resistance.

**3.16.8 Metabolic Pathway Analysis**
Metabolic pathway activity was assessed by calculating single-sample gene set enrichment scores for KEGG metabolic gene sets using the z-score method in the R package GSVA. Samples were then stratified into high- and low-expression groups based on the top and bottom 30% of gene expression, respectively. Differential activity of these metabolic pathways between the two groups was evaluated using the “limma” package.

**3.16.9 Immunological Subtype and Cell-Type-Specific Expression Analysis**
TCGA-LUAD samples were classified into six established immune subtypes based on previous literature[7] : C1 (wound healing), C2 (IFN-γ dominant), C3 (inflammatory), C4 (lymphocyte depleted), C5 (immunologically quiet), and C6 (TGF-β dominant). Tumors were then stratified into high- and low-PSMD14 expression groups using the median expression value as the cutoff. The distribution of these expression groups across the six immune subtypes was assessed for significant differences using the chi-square test.

Using the TISIDB database, we analyzed the expression of immunomodulators—including immunostimulators, immunosuppressants, chemokines, and major histocompatibility complex molecules—between PSMD14 high- and low-expression groups. Differential expression was assessed using the Wilcoxon rank-sum test, and results were visualized in a heatmap depicting the average expression of each gene per group.

**3.16.10 Cancer-Immunity Cycle Profiling**
The anticancer immune response was evaluated using the Tracking Tumor Immunophenotype (TIP) platform, which integrates ssGSEA and CIBERSORT to quantify immunophenotypic activity across the seven-step cancer-immunity cycle[8]. These steps include: release of cancer cell antigens (Step 1), cancer antigen presentation (Step 2), priming and activation (Step 3), immune cell trafficking to tumors (Step 4), immune cell infiltration into tumors (Step 5), T cell recognition of cancer cells (Step 6), and killing of cancer cells (Step 7). Spearman correlation analysis was applied to assess the relationship between PSMD14 expression and TIP activity scores, as well as inter-step correlations among TIP scores. Results were visualized using the linkET package.

**3.16.11 Single-Cell Expression Analysis**
Using the NSCLC_GSE117570 dataset, we classified all cell subpopulations into PSMD14-positive and PSMD14-negative groups based on detectable expression. The proportional contribution of each cell subtype to the PSMD14-positive population was calculated to identify cell types predominantly expressing PSMD14.

**3.16.12 Immune Signature Profiling**
LUAD patients were stratified into quartiles (Q1–Q4) based on gene expression levels, with Q1 and Q4 representing the highest and lowest 25% of expressors, respectively. Following the methodology established by Thorsson et al.[9], the mean score for each immune signature was computed across the four patient groups (missing values were excluded). The resulting data matrix was visualized as a heatmap using the R package “pheatmap”.

**3.16.13 Correlation Analysis**
Pearson correlation analysis and Fisher’s exact test were employed to assess the relationship between PSMD14 and HMMR gene expression. Using the cor.test function in R, we further evaluated correlations between PSMD14 expression and several genomic instability markers, including aneuploidy, homologous recombination defects, nonsilent and silent mutation rates, and SNV neoantigen load[7, 9]. The resulting correlation coefficients were visualized in a radar plot generated with the fmsb package.

**3.16.14 Single-Cell Expression and Cell Cycle Analysis**
Single-cell RNA-seq data from the GSE146773 dataset were derived from 1,152 individual U2OS FUCCI cells sorted by FACS and processed using SMART-seq2 chemistry. This enabled simultaneous quantification of transcriptomic profiles and cell cycle phases in individual cells. Normalized gene expression values were aligned along a linear pseudotime trajectory based on fluorescence intensity of cell cycle markers. Expression values were Z-score transformed via the scale function, and outliers beyond ±3 were excluded. Differences in gene expression across cell cycle phases were assessed using the Kruskal–Wallis rank sum test.

**Reference:**

1. Lee, E., et al., *Inferring pathway activity toward precise disease classification.* PLoS Comput Biol, 2008. **4**(11): p. e1000217.

2. Yuan, H., et al., *CancerSEA: a cancer single-cell state atlas.* Nucleic Acids Res, 2019. **47**(D1): p. D900-d908.

3. Hänzelmann, S., R. Castelo, and J. Guinney, *GSVA: gene set variation analysis for microarray and RNA-seq data.* BMC Bioinformatics, 2013. **14**: p. 7.

4. Liu, C.J., et al., *GSCA: an integrated platform for gene set cancer analysis at genomic, pharmacogenomic and immunogenomic levels.* Brief Bioinform, 2023. **24**(1).

5. Lapuente-Santana, Ó., et al., *Interpretable systems biomarkers predict response to immune-checkpoint inhibitors.* Patterns (N Y), 2021. **2**(8): p. 100293.

6. Schubert, M., et al., *Perturbation-response genes reveal signaling footprints in cancer gene expression.* Nat Commun, 2018. **9**(1): p. 20.

7. Thorsson, V., et al., *The Immune Landscape of Cancer.* Immunity, 2019. **51**(2): p. 411-412.

8. Xu, L., et al., *TIP: A Web Server for Resolving Tumor Immunophenotype Profiling.* Cancer Res, 2018. **78**(23): p. 6575-6580.

9. Thorsson, V., et al., *The Immune Landscape of Cancer.* Immunity, 2018. **48**(4): p. 812-830.e14.
