# Supplementary figures and images for: PSMD14 drives lung adenocarcinoma progression through HMMR stabilization and dual activation of TGF-β/Smad and PI3K/AKT/mTOR signaling
Source: Front Immunol. 2025 Dec 19;16:1720799. doi: 10.3389/fimmu.2025.1720799 (PMC12757429; doi:10.3389/fimmu.2025.1720799)

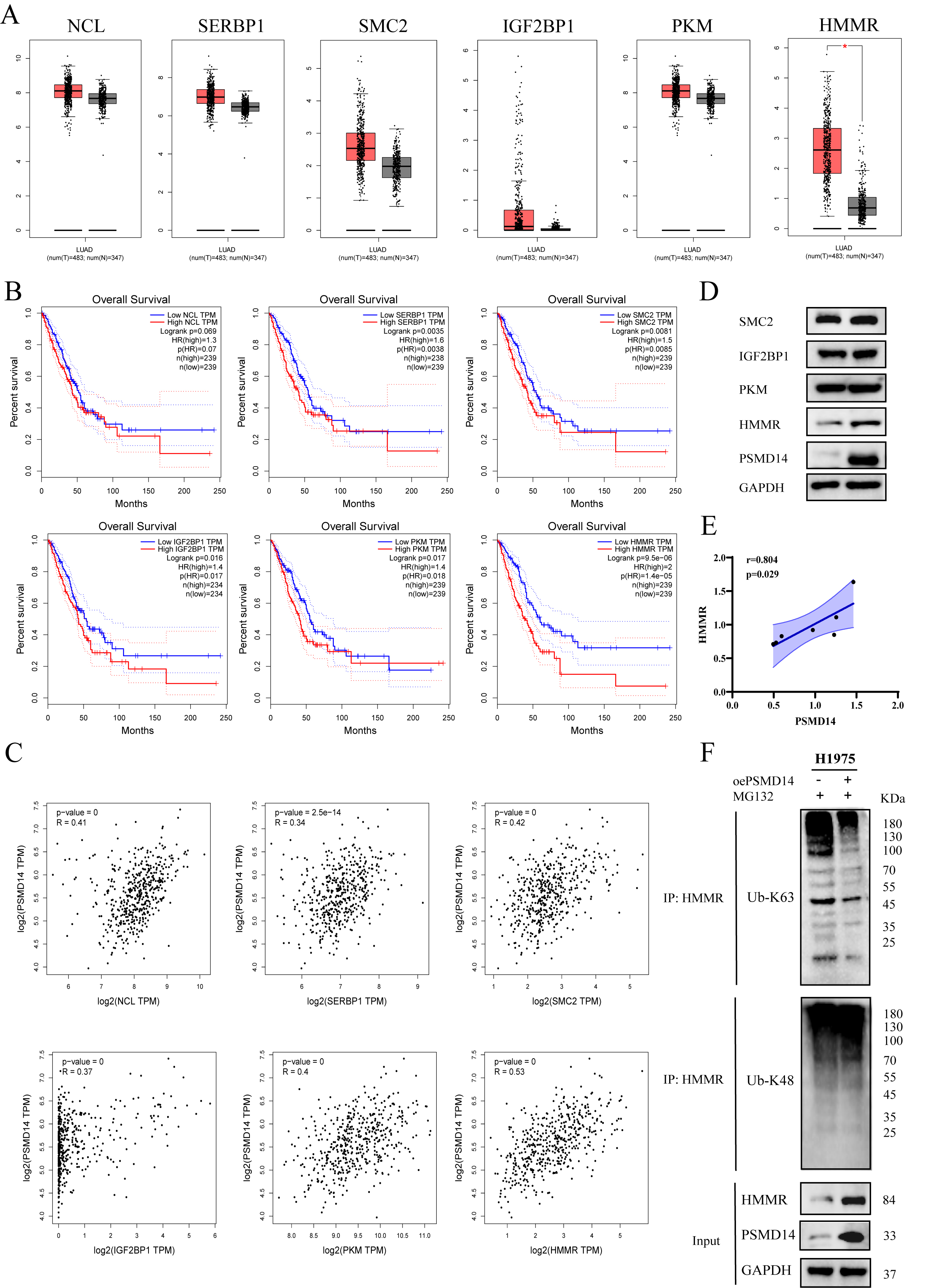

Supplement: Supplementary Figure 1 — Screening and validation of HMMR as a key downstream target of PSMD14 in LUAD. (A) mRNA expression levels of selected PSMD14-interacting proteins (NCL, SERBP1, SMC2, IGF2BP1, PKM, HMMR) in LUAD tumors and adjacent normal tissues from the GEPIA database. (B) Kaplan-Meier survival analysis of LUAD patients from the GEPIA database, stratified by high or low expression of the indicated genes. (C) Correlation analysis between PSMD14 and candidate gene expression in the TCGA-LUAD cohort. Pearson correlation coefficients are indicated. (D) Western blot analysis of the indicated proteins in LUAD cells after PSMD14 knockdown. (E) Scatter plot and statistical analysis of the correlation between PSMD14 and HMMR protein levels across different LUAD cell lines (Pearson r = 0.804, p = 0.029). (F) H1975 cells overexpressing PSMD14 were treated with MG132, and the ubiquitination of HMMR was assessed by immunoprecipitation using antibodies against K63-linked and K48-linked ubiquitin chains. [file DataSheet1.zip › Data sheet 1/Figure S1.jpeg]

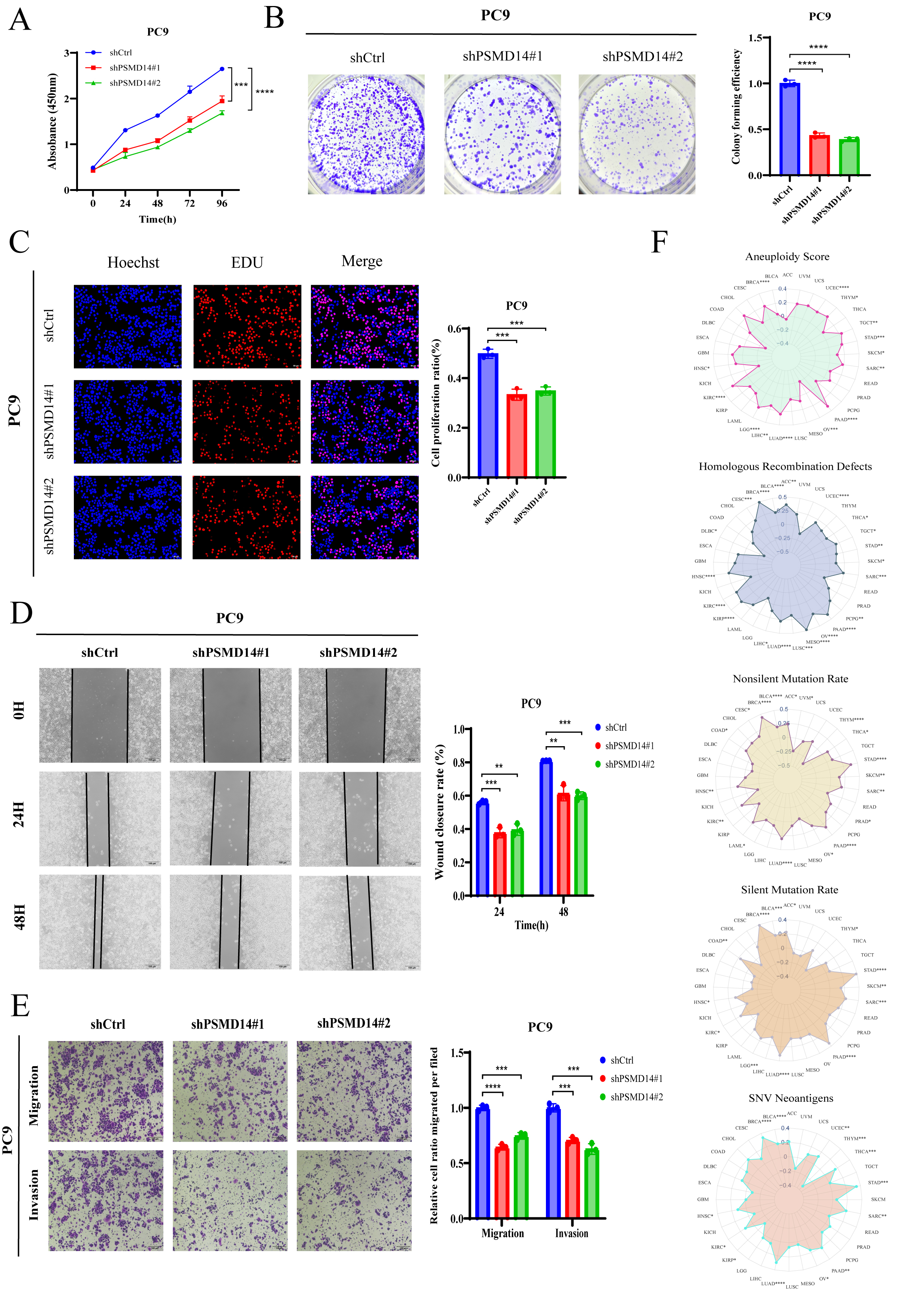

Supplement: Supplementary Figure 1 — Screening and validation of HMMR as a key downstream target of PSMD14 in LUAD. (A) mRNA expression levels of selected PSMD14-interacting proteins (NCL, SERBP1, SMC2, IGF2BP1, PKM, HMMR) in LUAD tumors and adjacent normal tissues from the GEPIA database. (B) Kaplan-Meier survival analysis of LUAD patients from the GEPIA database, stratified by high or low expression of the indicated genes. (C) Correlation analysis between PSMD14 and candidate gene expression in the TCGA-LUAD cohort. Pearson correlation coefficients are indicated. (D) Western blot analysis of the indicated proteins in LUAD cells after PSMD14 knockdown. (E) Scatter plot and statistical analysis of the correlation between PSMD14 and HMMR protein levels across different LUAD cell lines (Pearson r = 0.804, p = 0.029). (F) H1975 cells overexpressing PSMD14 were treated with MG132, and the ubiquitination of HMMR was assessed by immunoprecipitation using antibodies against K63-linked and K48-linked ubiquitin chains. [file DataSheet1.zip › Data sheet 1/Figure S2.jpeg]
